# Osteogenic Potential of 3D-Printed Porous Poly(lactide-*co*-trimethylene carbonate) Scaffolds Coated with Mg-Doped Hydroxyapatite

**DOI:** 10.1021/acsami.5c03945

**Published:** 2025-05-15

**Authors:** Mehmet Serhat Aydin, Carmen-Valentina Nicolae, Elisabetta Campodoni, Samih Mohamed-Ahmed, Masoumeh Jahani Kadousaraei, Mohammed Ahmed Yassin, Cecilie Gjerde, Monica Sandri, Izabela-Cristina Stancu, Ahmad Rashad, Kamal Mustafa

**Affiliations:** † Center of Translational Oral Research (TOR), Department of Clinical Dentistry, 1658 University of Bergen, 5009 Bergen, Norway; ‡ Advanced Polymer Materials Group, Faculty of Chemical Engineering and Biotechnologies, National University of Science and Technology Politehnica Bucharest, Bucharest 011061, Romania; § Institute of Science, Technology and Sustainability for Ceramics (ISSMC-CNR), Faenza, Ravenna 48018, Italy; ∥ Faculty of Medical Engineering, National University of Science and Technology Politehnica Bucharest, Bucharest 011061, Romania; ⊥ Bioengineering Graduate Program, Aerospace and Mechanical Engineering, University of Notre Dame, Notre Dame, Indiana 46556, United States

**Keywords:** polymeric scaffolds, magnesium doped-hydroxyapatite, microporosity, surface modification, bone tissue engineering

## Abstract

Extrusion-based 3D printing of thermoplastic polymers presents significant potential for bone tissue engineering. However, a key limitation is the frequent absence of filament porosity and the inherent osteoconductive properties. This study addresses these challenges by fabricating poly­(lactide-*co*-trimethylene carbonate) (PLATMC) scaffolds with dual-scale porosity: macroporosity achieved through controlled filament spacing and microporosity introduced via NaCl leaching. The inclusion of NaCl generated rough, porous surfaces that were well-suited for dip-coating with magnesium-carbonate-doped hydroxyapatite (MgCHA), thereby imparting osteoconductive functionality. Thermal analysis revealed that salt incorporation had minimal impact on the polymer’s thermal stability. Rheological studies and computational modeling indicated that NaCl reduced the viscosity under shear, leading to enhanced printability and faster extrusion speeds. After leaching, the scaffolds exhibited approximately 34% microporosity, which significantly increased water uptake and swelling capacity, despite the roughened surfaces slightly elevating hydrophobicity. The mechanical properties of PLATMC (with nonporous filaments) and p-PLATMC (with porous filaments) scaffolds showed a modulus of elasticity of 566 ± 118 and 101 ± 20 MPa, respectively, with strain values of 178 ± 54% and 84 ± 28%. Biological evaluations highlighted the compatibility of the p-PLATMC scaffolds. Cell viability and proliferation assays confirmed sustained cellular interaction over a 14 day period. Notably, alkaline phosphatase (ALP) activity was elevated in the porous scaffolds, and the MgCHA coating significantly enhanced mineral deposition by day 28, suggesting improved osteogenic potential. In conclusion, this study presents a robust strategy for fabricating 3D-printed PLATMC scaffolds with integrated filament porosity, offering a viable platform for osteoconductive coatings in bone tissue engineering applications.

## Graphical abstract

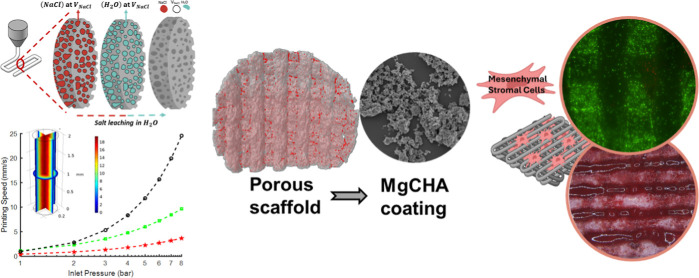

## Introduction

3D printing in bone tissue engineering (BTE) provides precise, patient-specific implants and scaffolds that can facilitate enhanced bone integration and regeneration. In recent years, polymers with aliphatic ester bonds that degrade through hydrolysis have been widely studied for their applications in BTE.^1,2^ Polycaprolactone (PCL) is one of the most widely used thermoplastic materials for scaffold fabrication due to its thermal stability, biocompatibility, and biodegradability.^3^ However, pure PCL exhibits slow degradation kinetics due to hydrolysis of its ester bonds, potentially resulting in a prolonged artificial 3D environment at defect site.^4^ In addition, its hydrophobic surface may hinder cellular activity in vitro and induce a foreign body reaction characterized by fibrous tissue formation in vivo.^5^

Amorphous aliphatic polycarbonate polymers such as poly­(trimethylene carbonate) (PTMC) offer flexibility and rapid degradation properties; however, it has limited tensile and compressive strength in BTE.^6^ To overcome this limitation, PTMC has been blended with polylactic acid (PLA) at various ratios to form a copolymer known as poly­(lactide-*co*-trimethylene carbonate) (PLATMC) with more tailored mechanical and degradation properties.^7,8^ PLATMC also undergoes a degradation process catalyzed by the acidic end groups of lactic acid.^9^ Hassan et al. demonstrated that 3D-printed PLATMC scaffolds exhibited superior hydrophilicity and mechanical properties, particularly enhanced toughness and elongation, compared to PCL.^5^ Additionally, the study showed that PLATMC scaffolds promoted the secretion of the bone extracellular matrix (ECM) by human bone marrow-derived mesenchymal stromal cells (hBMSCs) in vitro. Furthermore, compared with PCL scaffolds, PLATMC supported direct-contact osteogenesis in a calvarial defect model in rabbits without inducing fibrous tissue formation.^5^

Porosity, pore size, and interconnectivity are pivotal factors that strongly influence scaffolds’ mechanical and biological functions.^10^ While there is a wide range of reported optimal porosity for BTE, emerging evidence suggests a pore size larger than 300 μm is necessary for vascularization and bone formation.^11–14^ This macroporosity facilitates crucial functions such as oxygen diffusion, nutrient exchange, waste removal, cell migration, angiogenesis, and tissue growth within scaffolds.^15^ Furthermore, increasing the specific surface area of scaffolds by microporosity (pore size smaller than 100 μm) provides additional sites for protein adsorption. It promotes the faster release of degradation products, facilitating the interaction between the scaffolds and cells.^15^ The porosity and the material composition of polymeric scaffolds influence not only their biological properties but also their mechanical behavior, affecting their degradation patterns. However, scaffolds with higher porosity tend to exhibit lower stiffness and strength than nonporous scaffolds.^16^ Additionally, the degradation rate can vary depending on the material’s porosity and chemical structure.^17–19^ Consequently, the porosity and choice of biomaterial play a crucial role in determining the overall biological performance of the scaffold.^11,20^

Numerous methods exist for introducing internal porosity on the micro scale into thermoplastic scaffolds, including techniques like solvent casting, particulate leaching, gas foaming, phase separation, freeze-drying, and electrospinning.^1^ Conventional methods alone frequently lack precise control over scaffold porosity.^20^ On the other hand, 3D extrusion-based printing offers a highly automated fabrication process capable of producing patient-specific scaffolds with controlled large-scale porosity.^21^ Nonetheless, a drawback of this method is the inherent solid nature of the printed filaments, which lack porosity. A recent study has shown that surface topography can be tailored using controlled laser-assisted microengraving after 3D printing without compromising the strength of scaffolds.^22^ However, this technique does not provide a well-interconnected 3D porosity network throughout the structure. Our previous work modified the porosity and stiffness of 3D-printed PCL scaffolds by combining 3D printing with salt leaching and induced phase separation. This approach enabled the production of highly porous and stretchable scaffolds designed to facilitate an immune-mediated bone healing mechanism. The method successfully fabricated 3D-printed filaments with multiscale interconnected porosity, which supported hBMSC proliferation and osteogenic differentiation.^23^

Another critical aspect in fabricating polymeric scaffolds for bone regenerative applications is the incorporation of osteoconductive cues such as hydroxyapatite (HA), which provides a bioactive surface that encourages the adhesion, proliferation, and differentiation of osteogenic cells.^24,25^ Although synthetic HA (Ca_10_(PO_4_)_6_(OH)_2_) is frequently employed in tissue engineering research, it does not fully replicate the mineral phase of natural bone. In native bone, HA is nonstoichiometric and is often substituted with various ions, including HPO_4_^2–^, CO_3_^2–^, Na^+^, and Mg^2+^.^26^ Ion-doped forms of HA have shown promise in enhancing biological performance by improving cellular responses and accelerating bone healing.^27^ Doping HA with magnesium (Mg^2+^) stands out for its direct or indirect stimulation of bone formation and resorption, especially in fostering the early stage proliferation of osteoblasts.^28^ A recent study on Mg-based implants has highlighted that the concentration of Mg^2+^ in the microenvironment could enhance bone mass around these implants as they gradually degrade.^29,30^ Hydroxyapatite (HA) can be introduced into the scaffold structure in several ways. One approach is through physical blending or incorporation of the polymer matrix. The study has reported that few discernible distinctions were noted in vitro between PLATMC and PLATMC/HA blends regarding hBMSC seeding efficiency or proliferation.^31^ Moreover, the incorporation of HA into the polymer matrix reduced the mechanical strength of PLATMC.^31^

In contrast to the well-documented use of 3D-printed porous PCL scaffolds for bone tissue engineering applications,^23,32^ the application of 3D-printed PLATMC scaffolds remains relatively underexplored. Among the limited number of existing studies,^5,31,33^ the incorporation of controlled porosity within the individual printed filaments has received little attention, with most efforts focusing on scaffolds composed of fully solid filaments.^5,31,33^ Moreover, surface functionalization of PLATMC scaffolds with osteoconductive biomimetic hydroxyapatite, especially using ion-doped nanoparticles, has not yet been reported. The present study aimed to address these gaps by combining extrusion-based 3D printing of PLATMC with a salt-leaching technique to introduce internal microporosity within the printed filaments. This approach enabled modulation of the scaffold’s physicochemical and biological properties. Additionally, the study investigated the feasibility of functionalizing the porous filament surfaces through dip-coating with magnesium-doped biomimetic hydroxyapatite (MgCHA) nanoparticles to enhance osteoconductivity. To assess the potential of this strategy, human bone marrow-derived mesenchymal stem/stromal cells (hBMSCs) were cultured on the fabricated scaffolds, and their biological responses were evaluated. Three scaffold types were investigated: nonporous PLATMC, porous PLATMC (p-PLATMC), and MgCHA-coated porous PLATMC (p-PLATMC-HA). As a comparative control, porous PCL (p-PCL) scaffolds were also included. All scaffolds were comprehensively characterized by their physical, chemical, thermal, and mechanical properties. Furthermore, in vitro studies were conducted to evaluate their biological performance with hBMSC, focusing on cell viability, proliferation, and mineral matrix formation.

## Materials and Methods

### Ink and Granule Preparation

Poly­(lactide-*co*-trimethylene carbonate) (PLATMC), a copolymer composed of 70% polylactide (PLA) and 30% poly­(trimethylene carbonate) (PTMC) with an average molecular weight of 100 kDa (Resomer, Evonik, Germany), was used for extrusion-based 3D printing. To prepare the polymer ink, PLATMC pellets were fully dissolved in chloroform at 40 °C for 4 h. The polymer was dissolved at a concentration of 20% w/v (10 g of PLATMC in 50 mL of chloroform), which was optimized to ensure complete dissolution of the pellets.

To introduce porosity, sodium chloride (NaCl) particles (Sigma-Aldrich/Merck) sieved to a particle size range of 40–90 μm were added to the PLATMC solution at a 1:1 (polymer/salt) weight ratio (i.e., 10 g of NaCl in 10 g of dissolved PLATMC in chloroform). The resulting PLATMC-NaCl composite ink was then cast into glass Petri dishes prefilled with absolute ethanol to facilitate solvent exchange and promote film formation. The solvent evaporated under ambient conditions, forming solid polymer-salt composite sheets. These dried sheets were subsequently cut into granules using scissors and dried under vacuum at 30 °C overnight to remove residual solvent. These resulting granules were used for thermal and rheological characterization as well as for 3D printing. For comparison, polycaprolactone (PCL; Sigma-Aldrich/Merck, USA, 45 kDa) pellets were dissolved in acetone at 40 °C for 4 h using the same 20% w/v concentration. The PCL solution was processed into granules by using the same procedure as described for PLATMC.

### Thermal Characterization: TGA and DSC

Thermal analysis of the granules prepared for 3D printing was performed prior to the 3D printing process to evaluate the intrinsic thermal properties of the material. Thermogravimetric analysis (TGA) was conducted using a Netzsch TG 209 F1 Libra instrument (Germany) in a controlled oxygen/nitrogen atmosphere. Approximately 5 mg of each sample (PLATMC, PLATMC-NaCl granules) was heated under a temperature of 25 to 700 °C, with a heating ramp of 10 °C min^–1^ and a flow rate of 20 mL min^–1^. Isothermal TGA was employed to determine the composite granules’ decomposition temperatures and quantify the residual mass remaining after thermal degradation.

Differential scanning calorimetry (DSC) was performed to assess key thermal transitions, including the glass transition temperature (*T*_g_), melting temperature (*T*_m_), crystallization behavior, and their associated enthalpies. DSC measurements were conducted using a Netzsch 204 F1 Phoenix instrument. Approximately 10 mg of each granule sample was placed in alumina crucibles and subjected to a thermal ramp from 20 to 250 °C at a heating rate of 10 °C/min, under a nitrogen atmosphere with a 20 mL/min flow rate. As a control group, PCL-NaCl granules were prepared and analyzed using the same conditions for both TGA and DSC.

### Rheology of Granules

The rheological behavior of the same granules was investigated using a rotational rheometer (Kinexus Pro, Malvern Instruments, Brussels, Belgium) equipped with a Peltier element for temperature control. Single-use parallel plates (upper plate diameter of 25 mm) were employed at a 0.5 mm gap. The materials (roughly 0.5 g/sample) were loaded onto the lower plate and heated to printing temperatures of 200 °C for PLATMC and PLATMC-NaCl for 30 min. After temperature stabilization, the viscosity of the polymers was measured while varying the shear rate from 0.01 to 1000 s^–1^. Their viscoelastic behavior was evaluated through dynamic oscillatory measurements at a constant frequency of 1 Hz, while changing the stress from 0.01 to 1000 Pa, to determine the linear viscoelastic region (LVR). Using a constant stress value extracted from the LVR, the frequency of the oscillations varied from 10 to 0.1 Hz. PCL-NaCl granules were used as a control and heated to a printing temperature of 130 °C with the same settings.

### Mathematical Modeling and COMSOL Flow Simulation

Mathematical modeling was performed using a custom MATLAB script, including curve fitting to experimental data and parameter estimation (R2023a, MathWorks, US). For this process, the initial 16 data points of viscosity versus strain corresponding to [0–10 1/s] were considered by using non-Newtonian flow models, namely, power law (eq 1) and Carreau model (eq 2), as given in equations below.

1 $$Power\;law:\;\mu_{\infty }={m\cdot \left(\frac{\mathrm{\gamma ̇}}{{\mathrm{\gamma ̇}}_{ref}}\right)}^{n-1}$$

2 $$Carreau:\;\mu =\mu_{\infty }+\left(\mu_{0}-\mu_{\infty }\right){\left[1+\lambda {\mathrm{\gamma ̇}}^{2}\right]}^{\frac{\eta -1}{2}}$$

For the power law model, variables *m* and *n* are scalars that can be assigned arbitrary values. ${\mathrm{\gamma ̇}}_{ref}$ is a reference shear rate typically set to a default of 1 s^–1^. *n* < 1 describes a shear-thinning (pseudoplastic) fluid. When *n* = 1, the expression corresponds to that of a Newtonian fluid, whereas for the Carreau model, γ̇ is the shear strain rate, μ_∞_ is shear viscosity at infinite shear rate, μ_0_ is the shear viscosity at zero shear rate, λ is a time constant representing the fluid’s relaxation time, and η is a power law index (often called the “power-law exponent”).

The parameters derived from mathematical modeling were input parameters in the COMSOL Multiphysics (ver. 6.1, Comsol Inc., Sweden) for the Carreau model within the polymer flow module. Specifically, the values of λ and η obtained from the mathematical model were applied as Carreau parameters in COMSOL to define the flow (fully developed flow) properties. Average printing speed through varying nozzle diameter (*d*: 0.2, 0.4, 0.6 mm) and length (L: 2, 4, 6 mm) at various inlet air pressures (1–8 bar) was simulated. Experimentally, a nozzle with a diameter of 0.6 mm and a fixed length of 2 mm was used in 3D printing. Volumetric flow and printing speed can be described by Hagen–Poiseuille and flow rate equations (eq 3a to 3c) as follows, where *Q*, ν, and μ denote volumetric flow, flow velocity, and viscosity of the liquid, respectively. Flow velocity (eq 4) is associated with the radius (*r*) and length (*l*).

3a $$Q=\nu \cdot \pi r^{2}$$

3b $$Q=\frac{P\cdot \pi r^{4}}{8\cdot \mu \cdot l}$$

3c $$\nu \cdot \pi r^{2}=\frac{P\cdot \pi r^{4}}{8\cdot \mu \cdot l}$$

4 $$\nu \approx \frac{r^{2}}{l}$$

### 3D Printing of Granules in Scaffold Fabrication

All of the granules were 3D printed using a 3D-Bioplotter (EnvisionTech, Germany). NaCl-incorporated PLATMC granules were melted at 200 °C within a metallic cartridge of the 3D-Bioplotter. The material was extruded through a nozzle with a diameter of 0.6 mm and a length of 2 mm, using air pressure ranging from 8 bar at the start of printing to 4 bar near the end. The average printing speed ranged from 5 to 10 mm/s. To leach the NaCl and generate pores, the printed scaffolds were submerged in a bath containing 70% ethanol and water for 4 h, followed by a 5 day washing period in deionized (DI) water under stirring. The scaffolds were printed square-shaped (20 × 20 mm) with four layers, each having a layer height/slicing of 400 μm (80% of nozzle diameter). The internal pattern featured a periodic layout at angles of 0/90°. The distance between filaments was set at 1.2 mm (center-to-center distance), with a shift of 0.6 mm introduced in the *X*–*Y* plane on the third and fourth layer to enhance cell seeding efficiency. Similarly, PCL-NaCl granules were extruded at 130 °C with the same settings as a control. The four scaffold groups investigated in this study were as follows: (1) nonporous PLATMC (PLATMC), (2) porous PLATMC (p-PLATMC), (3) MgCHA-coated porous PLATMC (p-PLATMC-HA), and porous PCL (p-PCL) as a control.

### Porosity and Structural Analyses of Scaffolds

The overall internal microporosity of scaffold filaments was evaluated by weighing three scaffolds under wet and dry conditions. Internal porosity induced by salt leaching can be found via the ratio of leached NaCl volume to scaffold volume (eq 5). The Supporting Information provides detailed calculations and steps (eqs S1–S11).

Microcomputed tomography (micro-CT, Bruker SkyScan 1172, Belgium) was employed to evaluate microporosity and size distribution. To visualize the NaCl-based porosity, midresolution (2k) micro-CT scans were conducted with a magnification of 10 μm. The scanning parameters for micro-CT are summarized in Table 1.

**Table 1:** Micro-CT Scanning Parameters Used in Porosity and Structural Analysis

type of scanning	camera pixel (μm)	resolution pixels	pixel size (μm)	voltage (kV)	exposure time (ms)	rotation step
2k	8.75	2000 × 1336	10	35	211	0.7

To investigate the surface topography and porosity, scaffolds were coated with a layer of gold-palladium using a sputter coater (DSR1, Vac Techniche, UK) and imaged with a scanning electron microscope (SEM; Zeiss Leo Supra VP 55, Jena, Germany) at an acceleration voltage of 10–15 kV and 5–7 mm working distance. Cross-sections of scaffolds were exposed by cutting them into liquid nitrogen using pliers to expose the internal structure and porosity.

5 $${Ø}_{\mu }=\frac{V_{salt}^{s}}{V_{polymer}^{s}+V_{salt}^{s}}$$

### Water Contact Angle, Absorbance, and Swelling

The surface wettability of 3D-printed samples (1-layer, flat sheet samples) was assessed by static contact angle measurements and performed with a Drop Shape Analyzer 100 (Krüss Scientific GmbH, Hamburg, Germany) using the sessile drop method. Droplets of distilled water (2 μL) were placed on the sample surface, and the water contact angle was monitored for 60 s after deposition at room temperature. The results were processed using the Young-LaPlace method with Advanced software (version 1.7.2.1) and expressed as an average of 5 measurements per sample. To ensure a flat surface for proper contact angle measurement, the sheet samples PLATMC and p-PLATMC with 1 layer were printed by extruding the filaments adjacent. Measurements (*n* = *5*) were performed on the dry samples, which had been previously washed. Similarly, absorbance and 1D swelling were measured through rehydrating dry-washed samples. The water absorbance and 1D swelling (%) were conducted through gravimetric analysis (eq 6) and photographic length measurement, respectively. For each group, three samples were punched (Ø = 8 mm) from 3D-printed square scaffolds. Before being leached (when dry), the scaffolds were weighed and photographed. This process was repeated both under wet and dry conditions after leaching. The difference in weight and length between the wet and dry scaffolds determined the water absorbance and 1D swelling percentage, respectively. Conversely, the subtraction in weight before and after leaching, under dry conditions, indicated the quantity of NaCl leached out, thus indicating NaCl-induced microporosity. The same procedure was applied to the control group of p-PCL.

6 $$Water\;absorbtion=\frac{W_{wet}^{s}-W_{dry}^{s}}{W_{dry}^{s}}$$

### Mechanical Tensile Testing

Tensile stress tests were performed to assess the mechanical properties of 3D-printed dog bone specimens (*n* = *3*) with dimensions of 0.3 mm thickness (1 layer), 5 mm width, and 10 mm gauge length. A uniaxial tensile load was applied in the printing direction. The tests were conducted by using a universal testing machine (MTS, 858 mini Bionix II instrument, Eden Prairie, MN, USA) at a strain rate of 0.1 mm/s.

### Synthesis and Characterization of MgCO_3_-Doped Hydroxyapatite

MgCO3-doped hydroxyapatite (MgCHA) was prepared according to the protocol reported by Landi et al.^34^ Briefly, it was synthesized through a neutralization method based on the simultaneous controlled dripping of 49.6 mL of 1.2 M H_3_PO_4_ solution (Sigma-Aldrich, 85% pure) and 46 mL of 0.8 M solution of NaHCO_3_ (Sigma-Aldrich) in 82.7 mL of 1.2 M of Ca­(OH)_2_ (Sigma-Aldrich, 95% pure) aqueous suspension containing 8.48 g of MgCl_2_·6H_2_O and maintained at 25 °C under magnetic stirring. The precipitated product was aged for 24 h at 25 °C, washed with deionized water through centrifugation three times, lyophilized, sieved at 150 μm, and then micronized at 3 μm.

MgCHA powder morphology was assessed by electron scanning microscopy (SEM, Carl Zeiss Sigma NTS Gmbh Öberkochen, Germany). Sample preparation for morphological evaluation included powder fixation onto aluminum stubs by carbon tape, followed by Au coating applied by sputtering (QT150T, Quorum Technologies Ltd., UK).

To determine MgCHA’s chemical composition, inductively coupled plasma-optical emission spectrometry analysis (ICP-OES 5100, vertical dual view apparatus, Agilent Technologies, Santa Clara, CA, USA) was performed. In brief, 10 mg of MgCHA was dissolved in 50 mL of a 2 wt % HNO_3_ solution before the analysis.

The XRD pattern was obtained by using a D8 Advance diffractometer (Bruker, Karlsruhe, Germany) equipped with a Lynx-eye position-sensitive detector. The analysis employed Cu Kα radiation (λ = 1.54178 Å) at 40 kV and 40 mA. Spectra were recorded in the 2θ range from 20° to 80°, with a step size (2θ) of 0.02° and a counting time of 0.5 s.

Thermogravimetric analysis (STA 449 F3 Jupiter instrument, Netzsch, Geraetebau, Germany) was used to calculate the residual mass of MgCHA, which can be used to calculate its carbonation wt % indirectly. The analysis was conducted in alumina crucibles from room temperature to 1100 °C, at a heating rate of 10 °C/min under a nitrogen flow. The sample weighed approximately 10 mg.

Attenuated total reflection Fourier transform infrared spectroscopy (ATR-FTIR) served as proof of hydroxyapatite identity and to prove the actual carbonation of MgCHA. The analysis was done with a Nicolet 5700 spectrometer (Thermo Fisher Scientific Inc., Waltham, MA, USA) in ATR mode (FTIR-ATR) using an ATR iD7 accessory. Instrumental resolution was set up to 4 cm^–1^, and 16 scans were collected per sample from 4000 to 400 cm^–1^.

### Dip-Coating of MgCHA on Porous PLATMC

Porous PLATMC (p-PLATMC) scaffolds underwent a surface functionalization process through a dip-coating method with MgCHA. Various liquids, compositions, and concentrations in the resuspension solution were optimized to ensure optimal surface coverage and coating efficiency. Initially, to decide solvent/liquid, samples were coated with 5% MgCHA using both DI water and ethanol, at different compositions ranging from 100% DI water and 100% ethanol to various mixtures at intervals of 70–30%, 50–50%, and 30–70% water–ethanol. Subsequently, samples were coated with different concentrations (1%, 2.5%, and 5 wt %/wt) of MgCHA in absolute ethanol for 2 h. 1% resuspension concentration was decided to be used in further experiments. The coating coverage was qualitatively characterized by SEM and micro-CT and quantitatively by sample weight measurement.

## Scaffold-hBMSC Interactions

### Isolation and Expansion of hBMSC

The cytocompatibility and osteogenic performance of the printed scaffolds were evaluated with hBMSC that were isolated and characterized under ethical approval from the Regional Committee for Medical and Health Research Ethics in Norway (2020/7199/REK sør-øst C).^35^ Cells (from passage 2 to 4) were expanded in a growth medium (Alpha-Minimum Essential Medium, α-MEM, Gibco, ThermoFisher Scientific) supplemented with 10% fetal bovine serum (FBS, HyClone, GE Healthcare, Utah, USA) and 1% antibiotics (100 U/mL penicillin and 0.1 mg/mL streptomycin) (Gibco, ThermoFisher Scientific) at 37 °C and 5% CO_2_. For osteogenic differentiation of the cells after seeding on the scaffolds, the growth medium was supplemented with 10 mM β-glycerophosphate (500 μL/100 mL), 10 nM dexamethasone (20 μL/100 mL), and 0.05 mM l-ascorbic acid 2-phosphate (35 μL/100 mL) (Sigma-Aldrich).

### Viability and Proliferation of hBMSC Seeded onto 3D Scaffolds

To ensure proper fitting within 48-well low adherent plates (Sarstedt, Numbrecht, Germany), disc-shaped scaffolds (Ø = 8 mm) were punched out of the printed squares. The punched scaffolds were sterilized using 70% ethyl alcohol for 1 h before UV sterilization for 30 min. Sterilized scaffolds were prewetted overnight in the growth medium before seeding cells at a density of 1 × 10^5^ cells/scaffold in 50 μL cell suspension. After 1.5 h of initial incubation, wells were supplemented with 750 μL of media.

To assess cell viability, live/dead (ThermoFisher Scientific) staining was employed on days 1, 7, and 14. After washing with PBS, samples were incubated for 40 min at RT in the dark in a PBS solution containing EthD-1 and Calcein-AM. Subsequently, the scaffolds were washed with PBS and imaged using a fluorescence microscope (Nikon, Eclipse 80i, Tokyo, Japan).

Furthermore, PrestoBlue (PB) assay was employed to evaluate the metabolic activity and viability of hBMSC on the scaffolds. On days 1, 7 and 14, cell-seeded scaffolds (*n* = 4) were transferred to a new 48-well plate, and fresh medium with 10% (v/v) ready-to-use PrestoBlue solution (Invitrogen, ThermoFisher Scientific) was added to each well, followed by a 15 min incubation at 37 °C. The fluorescence (550–590 nm) was then measured using a microplate reader (VarioskanTM LUX, ThermoFisher Scientific).

For proliferation assessment, the Quant-iTTM PicoGreen DNA kit (Invitrogen, ThermoFisher Scientific) was utilized. On each point, scaffolds (*n* = 4) were washed with PBS, treated with 0.1% Triton-X/PBS, and stored at – 80 °C. After two freezing–thawing cycles and 40 s of sonication, 50 μL of each sample and an equal amount of working PicoGreen solution were combined in a 96-well according to the manufacturer’s protocol. Fluorescence at 480/520 nm was measured using the microplate reader.

### Mineralization Assessment of hBMSC Seeded onto Scaffolds

Alkaline phosphatase (ALP) activity was analyzed from the same Triton- X 100 lysates used for the proliferation test (*n* = 4) using a commercial kit (p-Nitrophenyl Phosphate Liquid Substrate System, P7998-100 mL, Sigma-Aldrich/Merck). Briefly, equal volumes of ALP working solutions and samples (50 μL) were pipetted into the wells of a 96-well plate and incubated for 30 min at 37 °C. Absorbance at 405 nm was measured using the microplate reader.

On day 28 of culture in an osteogenic medium, Alizarin Red-S staining (Sigma-Aldrich) was used to detect the calcium deposition of the hBMSC. The cultured cells on the scaffolds (*n* = 4) were fixed with 4% paraformaldehyde for 15 min and then stained with 2% Alizarin Red-S solution (pH 4) for 30 min at RT. After several washes with Milli-Q water, the scaffolds were air-dried and imaged using a stereomicroscope (Leica M205C, Wetzlar, Germany). To quantify the staining, samples were incubated in 1 mL of 100 mM cetylpyridinium chloride (CAS 6004-24-6, Sigma-Aldrich/Merck), and the absorbance was measured at 540 nm. Scaffolds without cells were stained to serve as the controls.

### Ion Release via Inductively Coupled Plasma Optical Emission Spectrometry

Inductively coupled plasma optical emission spectrometry (ICP-OES) analysis was performed to determine the presence and concentration of calcium (Ca), phosphorus (P), and Mg ions in cell culture media collected from the control group (p-PLATMC) and the target group (p-PLATMC-HA) at both initial and final time points of cell culture. Approximately 500 μL of osteogenically supplemented media from each sample was collected in Eppendorf tubes. These samples were diluted 20-fold for ICP-OES analysis. The diluted samples were analyzed using an ICP-OES (iCAP 7600 ICP-OES Analyzer, Thermo Fischer) with the detection limit of 0.02, 0.03, and 0.01 (mg/L) for Ca, Mg, and P, respectively. The SPS-SW2 (surface water) was a certified reference material. An ICP Calibration/Quality Control Standard was used (10 ppm of 43 Element IV-ICPMS-71A-125 ML, Inorgenic Ventures, Christiansburg, VA, USA).

### Statistical Analysis

Statistical analysis was conducted using one-way ANOVA with Tukey’s multiple comparison test between the groups and two-way ANOVA between the time points using GraphPad (version 5, California, USA). The sample size has been specified in the relevant sections of the Materials and Methods and the corresponding figure legends. Data are expressed as the mean ± the standard deviation (SD). Differences were considered statistically significant at *p* < 0.05*.*

## Results

### Porous PLATMC Scaffolds Were Successfully 3D Printed

Polymer inks were prepared by dissolving PLATMC in chloroform with or without NaCl particles, respectively. The polymer pellets were then successfully printed at a high temperature (Figure 1a,b). By introducing the distance between the printed filaments as a design parameter of 3D printing, bulk macroporosity (open channels) was created (Figure 1c,d). The printed PLATMC-NaCl scaffolds displayed considerable swelling and water absorbance after salt leaching and washing (Figure 1c) and exhibited foldable and shapeable features (Figure 1d). Nonporous PLATMC showed promising buildability (Figure 1e,f). Notably, while nonporous PLATCM was transparent, porous PLATMC displayed opaque optical properties (Figure 1g). The micro-CT 3D model illustrated the presence and distribution of the introduced large microporosity throughout the p-PLATMC scaffolds after leaching out NaCl particles (Figure 1h).

**Figure 1:**
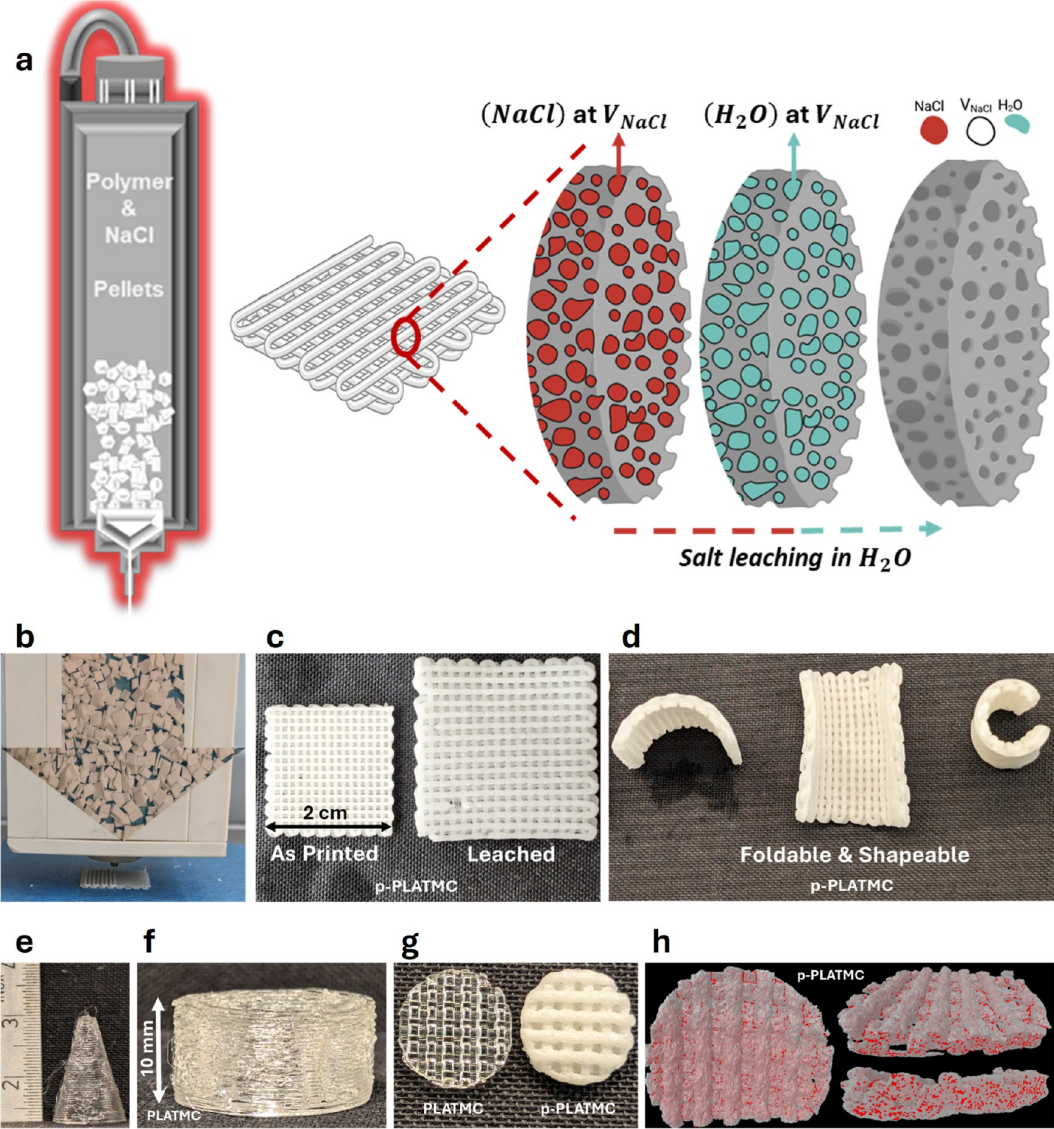
(a) 3D printing of composite granules. (b) Image depicting high-temperature (HT) printing through melting of composite granules. (c) Comparison of scaffolds before and after leaching, along with their swelling and water absorption capabilities. (d) Foldability and flexibility demonstration of p-PLATMC scaffolds. (e,f) Buildability test of PLATMC. (g) Optical property comparison between nonporous and porous PLATMC. (h) 3D pore structure model of p-PLATMC using micro-CT imaging.

### Thermal Properties and Composition Analyses of Granules

Thermogravimetric analysis (TGA) and differential scanning calorimetry (DSC) were employed to examine the thermal characteristics of PLATMC and PLATMC-NaCl granules used in 3D printing, with results shown in Figure 2 and material thermal properties summarized in Table 2. The findings indicated that adding salt (NaCl) to the PLATMC did not notably alter the thermal properties of the polymer. The decomposition temperatures of PLATMC and PLATMC-NaCl were comparable; they were lower than those of the control granules of PCL-NaCl (Figure 2a). TGA performed under nitrogen and air atmospheres showed similar trends, although slightly higher residual masses were recorded under air conditions. The observed residual was approximately the initial NaCl percentage in the composition, which was 50%.

**Figure 2:**
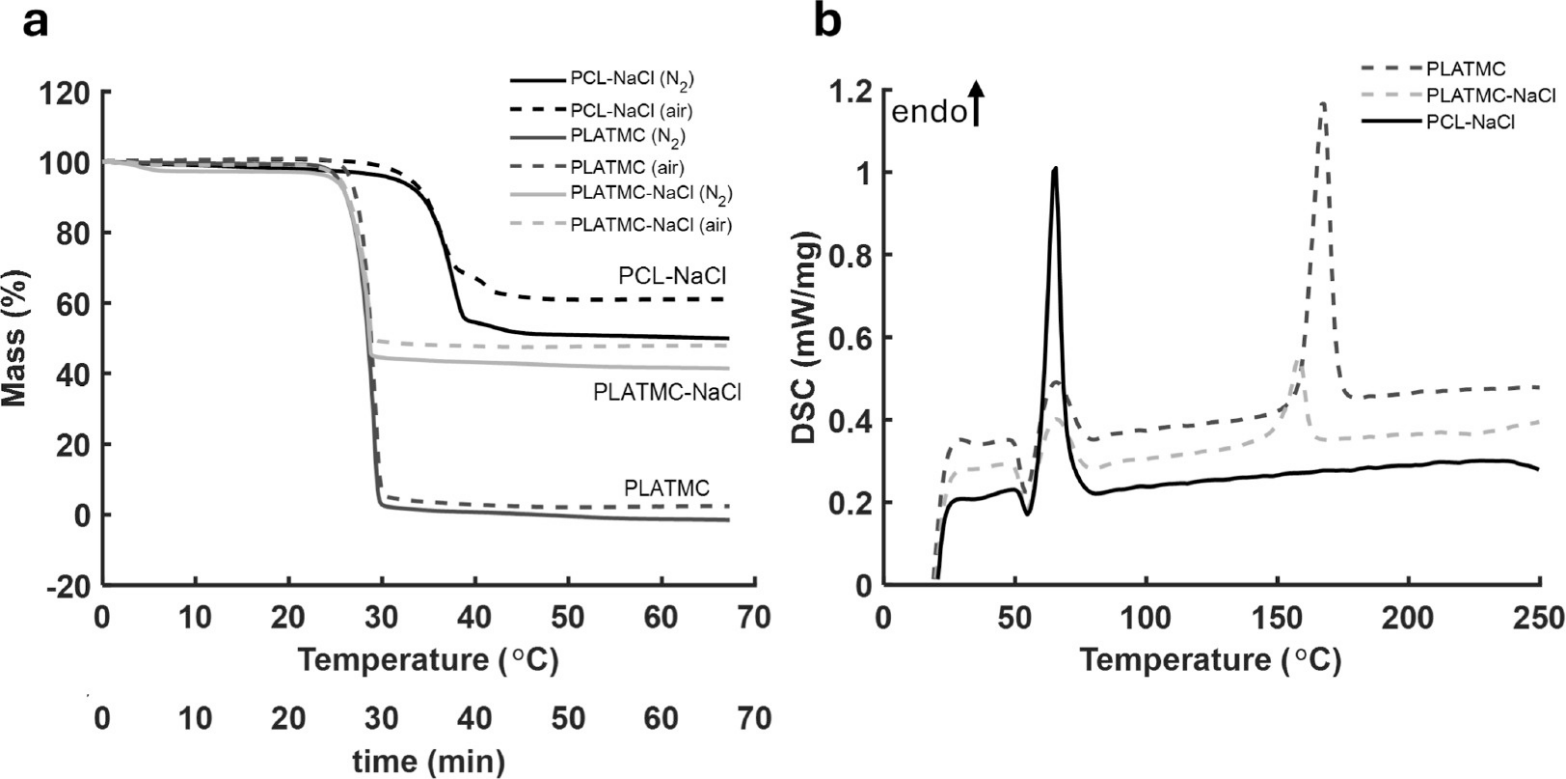
(a) Thermogravimetric analysis (TGA) showing mass change (%) and decomposition of custom granules conducted under nitrogen (N_2_) and air (b) differential scanning calorimetry (DSC) analysis displaying the melting temperature of the granules used in 3D printing.

**Table 2:** TGA and DSC Analyses Show the Custom Granules’ Thermal Properties in 3D Printing^a^

analysis	property	PLATMC	PLATMC-NaCl	PCL-NaCl
TGA	residual (%)	0	41.44	49.95
	*T*_d_ (°C)	310.1	302.3	372.0
DSC	*T*_g_ (°C)	55.2	55.1	–63.3
	*T*_m_ (°C)	167.4	158.1	65.1
	*C*_p_ J/(g K)	1.025	1.284	0.086
	Δ*H*_m_ (J/g)	37.86	10.97	29.12
	crystallinity	high	low	moderate
experimental	*T*_p_ (°C)	200	200	130

a *T*_g_, *T*_m_, *T*_d_, and *T*_p_ denote glass transition, melting, decomposition, and printing temperatures, respectively.

DSC analysis was performed on PLATMC, PLATMC-NaCl, and PCL-NaCl granules over a temperature range of 20 to 300 °C (Figure 2b). All samples exhibited endothermic transitions. For PLATMC and PLATMC-NaCl, two primary endothermic peaks were observed. While the first, low temperature peak, appeared near 65–70 °C, the second, more prominent high-temperature endothermic peak, occurred between 150 and 180 °C. This peak, indicating the melting temperature of the copolymer, occurred at 167.4 °C for PLATMC and 158.1 °C for PLATMC-NaCl. The associated melting enthalpy (Δ*H*_m_) values were 37.86 and 10.97 J/g, respectively, indicating a decrease in crystallinity due to the presence of NaCl. The glass transition temperatures (*T*_g_) were also identified in both PLATMC samples. PLATMC showed a *T*_g_ onset at 55.2 °C with a heat capacity change (Δ*C*_p_) of 1.025 J/(g K), while PLATMC-NaCl had a *T*_g_ onset at 55.1 °C with a slightly higher Δ*C*_p_ of 1.284 J/(g K). In contrast, the PCL-NaCl-controlled sample displayed a single endothermic peak at 65.1 °C, corresponding to the melting of PCL. No glass transition was observed for PCL-NaCl within the measured temperature range. However, a separate DSC analysis between −70 and 20 °C recorded the glass transition at −63.3 °C. DSC results also revealed that both PLATMC and PLATMC-NaCl exhibited considerably higher melting temperatures than PCL-NaCl, which has implications for the required processing and higher printing temperature.

### Rheology and Modeling Helped to Simulate Polymer Extrusion

Rheological analysis was conducted to investigate the flow behavior of PLATMC and its salt-containing composites (PLATMC-NaCl) to simulate the extrusion behavior during 3D printing. Viscosity measurements as a function of shear rate showed that pure PLATMC maintained a high viscosity, remaining in the range of thousands of Pa.s even at the upper end of the shear rate spectrum (10 s^–1^). In contrast, the addition of NaCl significantly reduced viscosity under shear for both PLATMC–NaCl and the PCL–NaCl control, indicating enhanced shear-thinning behavior and improved processability.

Curve fitting of the rheological data using both the power law and Carreau models demonstrated good agreement with the experimental values. While both models accurately described the non-Newtonian behavior of the materials, the Carreau model provided a slightly better fit at higher shear rates, particularly for PLATMC and PLATMC-NaCl (Figure 3a). The fitted parameters derived from the models are summarized in Table 3.

**Figure 3:**
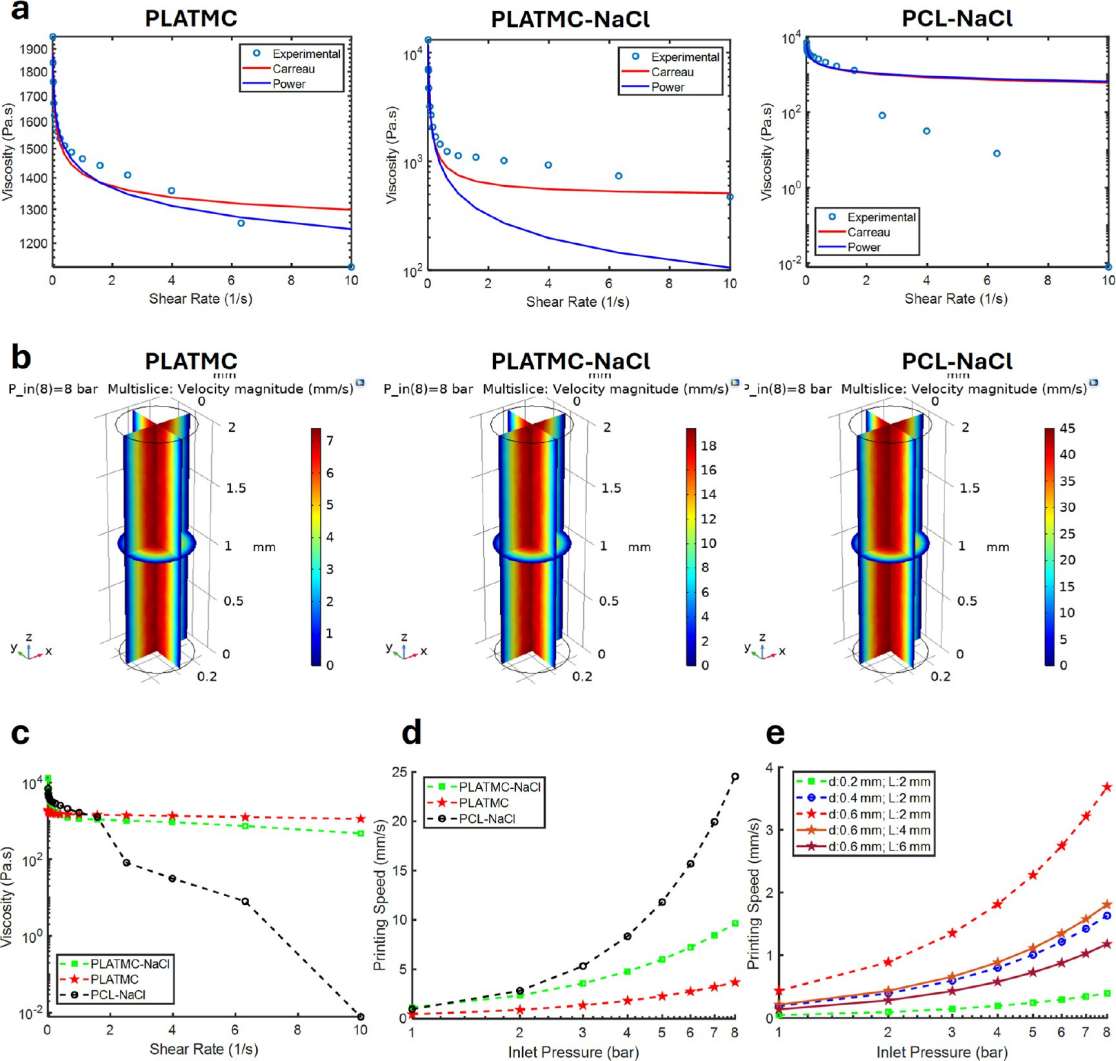
(a) Mathematical model and curve fitting (power and Carreau models) applied to the rheological viscosity data. (b) COMSOL simulation illustrates the velocity profile of polymer flowing through a 0.6 mm nozzle under a maximum pressure of 8 bar. (c) Viscosity versus shear rate plot for polymer groups, demonstrating shear thinning behavior. (d) Average printing speed through the nozzle at various inlet air pressures. (e) Average printing speed of PLATMC through varying nozzle diameter (*d*: 0.2, 0.4, 0.6 mm) and length (*L*: 2, 4, 6 mm) at various inlet air pressures (1–8 bar).

**Table 3:** Curve Fitting Parameters of Carreau and Power Models through Mathematical Modeling Based on Rheological Properties

	parameters					
model	Carreau			power		
groups	λ: relaxation time (s)	η: power index	*R* ^2^	*m*: consistency coefficient (Pa s)	*n*: power index	*R* ^2^
PLATMC	107	0.77	0.92	1424	0.94	0.94
PLATMC-NaCl	87	0.14	0.93	508	0.32	0.96
PCL-NaCl	92	0.64	0.93	1400	0.66	0.93

The maximum flow velocity is observed at the center of the nozzle, where shear stress is minimized but dynamic viscosity is maximized (Figure 3b). In contrast, near the nozzle wall, where shear stress peaks, the flow velocity is close to zero (Figures 3b and S1). As seen in the viscosity versus shear rate results (Figure 3c), PLATMC-based melts have a lower printing speed than the control (PCL-NaCl). At pressures up to 2 bar, all materials face extrusion difficulty, with no notable differences in the printability speeds of the polymers. However, at 8 bar, higher pressures resulted in higher printing speeds, approximately 4 mm/s for PLATMC and 10 mm/s for PLATMC-NaCl. Printing speed at that pressure was around 25 mm/s for the control (PCL-NaCl; Figure 3d). As expected, the addition of NaCl gave a rise in initial/zero shear viscosity. However, the presence of NaCl decreased viscosity with shear, even at both lower and higher shear rates, after the material was subjected to the shear force, placing it below that of nonporous PLATMC on the graph. Average printing speed of PLATMC through varying nozzle diameter (*d*: 0.2, 0.4, 0.6 mm) and length (*L*: 2, 4, 6 mm) at various inlet air pressures (1–8 bar) revealed that a larger nozzle diameter resulted in a higher printing speed, while a longer nozzle length led to a reduction in printing speed (Figure 3e).

### Microporosity Effect on Structural Properties and Surface Roughness

Washing scaffolds to leach NaCl out yielded inherently porous scaffolds, as proven by micro-CT (Figure 4). The expected total microporosity from salt leaching was theoretically calculated as 34.1% (salt to polymer ratio is 1). This calculation was based on the volume-weight-density relationship for NaCl. The total actual microporosity (measured experimentally) of p-PLATMC was 33.8 ± 0.2%, whereas that of the control (p-PCL) was 33.5 ± 0.1%. The total microporosity of p-PLATMC was calculated via micro-CT as 37.4 ± 1.8%, whereas that of the control (p-PCL) was 34.7 ± 3.6%. The p-PLATMC exhibited an average wall thickness of 35 ± 4 μm and a micropore size of 37 ± 15 μm, while the control had values of 51 ± 16 μm and 45 ± 15 μm, respectively. The macroporosity due to the distance between the filaments in 3D printing design was roughly calculated via micro-CT as 25–30%. The microporosity measurements obtained through gravimetric measurement and CT scans were statistically similar. Furthermore, these results did not differ significantly from the theoretical predictions. Additionally, the 3D reconstructed image in CT revealed that the created porosity is evenly distributed throughout the scaffold (Figure 4).

**Figure 4:**
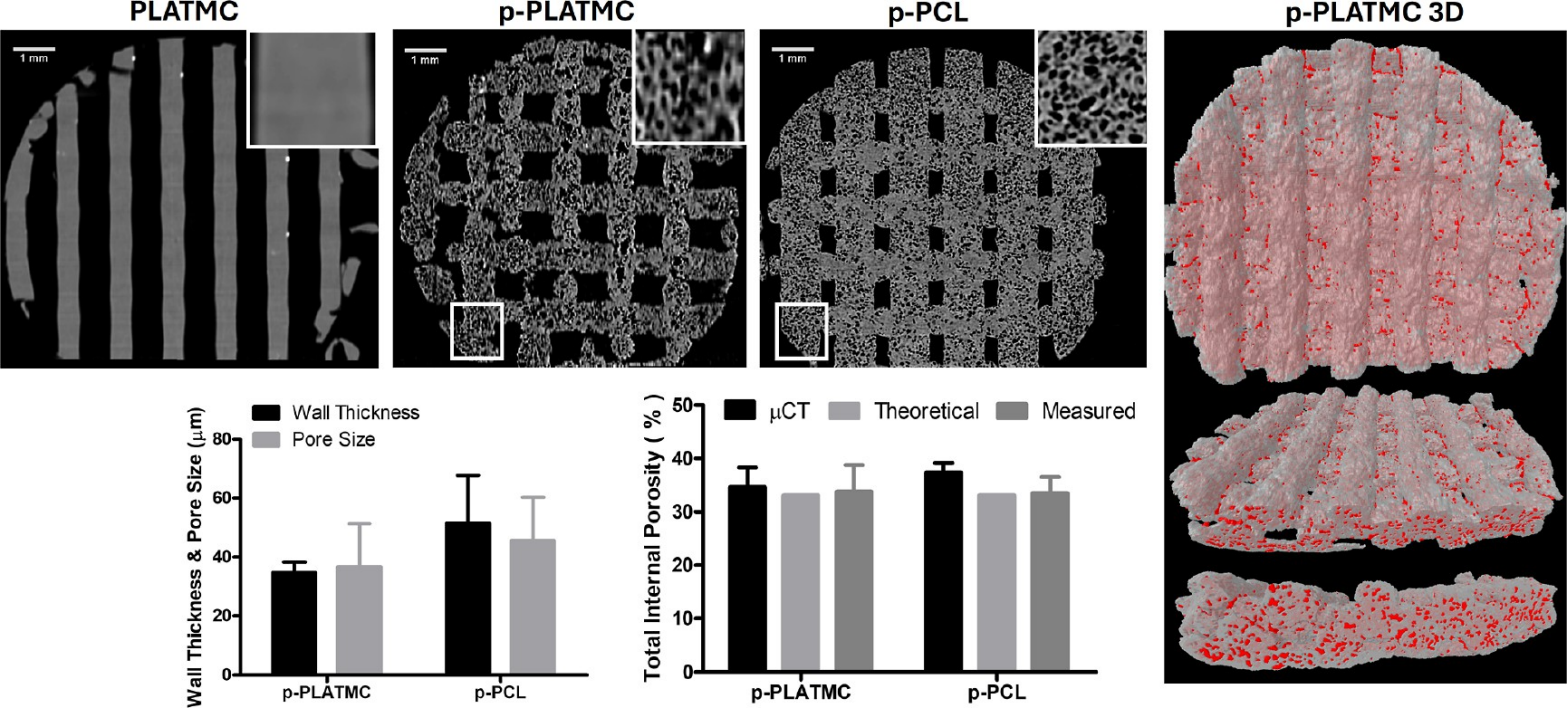
Micro-CT 3D morphological analysis of 3D-printed porous scaffolds, providing both qualitative and quantitative evaluation of pores generated by NaCl leaching (*n* = 3)*.*

Scanning electron microscopy images of scaffolds post-washing are depicted in Figure 5. Examination of filament cross-sections revealed that the introduction of internal porosity depends on the presence of NaCl. As anticipated, conventional extrusion-based 3D printing of PLATMC without NaCl yielded solid, nonporous filaments. Moreover, the surface characteristics (including porosity and roughness) of printed scaffolds were influenced by salt leaching. Scaffolds from the PLATMC group without added salt exhibited a smooth, nonporous surface, whereas those with leached salt displayed a rough, porous surface. Furthermore, upon comparing the porous PLATMC group with the control (p-PCL) group, it was observed that despite employing the same fabrication technique, there were disparities in surface properties. Introducing porosity to PLATMC led to rougher surface characteristics, aligning more closely with its cross-section, in contrast to the p-PCL control. In summary, SEM images corroborated the previously observed and quantified findings from micro-CT analysis.

**Figure 5:**
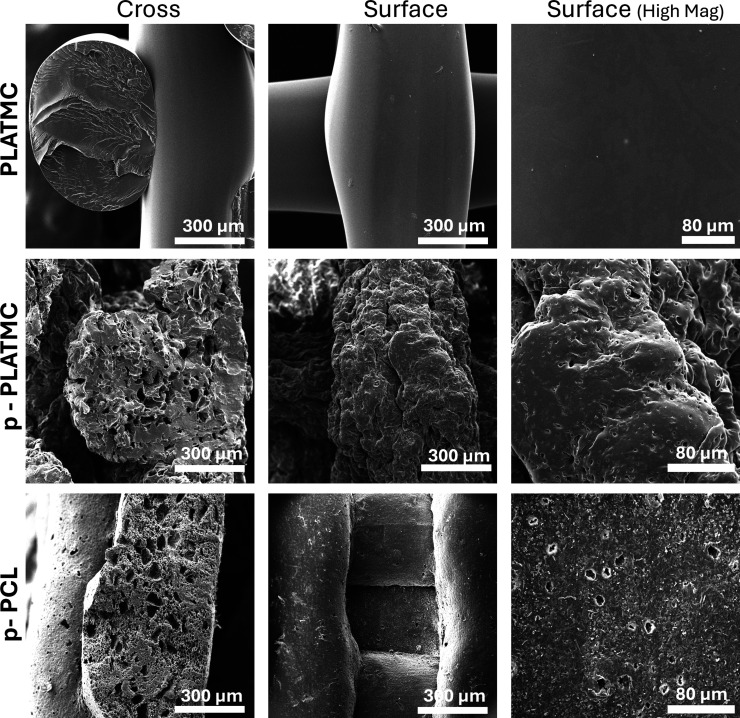
Scanning electron microscopy (SEM) of 3D-printed scaffolds showing cross-section and surface morphology, topography, and roughness.

### Porosity and Roughness Impact on Water–Material Interaction

Contact angle (CA) measurements on dry samples showed that PLATMC has the highest hydrophilic surface (lowest avg. CA) when the first-time water interacts with the dry surface at the contact moment (*t* = 0). Also, the CA of PLATMC decreases faster than other groups with time, especially within 10 s (Figure 6b,c). Furthermore, microporosity induction in the polymers revealed that there is an increase in CA, which makes them more hydrophobic. Similarly, the SEM images revealed increased surface roughness (Figure 5). The difference in CA is less pronounced between p-PLATMC and the control (p-PCL). The porous form of PLATMC absorbs more water than its nonporous form and the control (p-PCL). While nonporous PLATMC can absorb water approximately 16 wt %, its porous counterpart can absorb about 190 wt % (Figure 6d). The control, on the other hand, can absorb around 45 wt.%. The same trend is valid for the swelling characteristics of scaffold groups after they were leached and dried first, then leached and dried again. Then they were soaked in water (Figure 6e). The swelling ratio based on water absorbance/uptake is the highest for p-PLATMC. However, it was observed that 1D length change can go up to 50 wt % for p-PLATMC while they are leached to remove NaCl.

**Figure 6:**
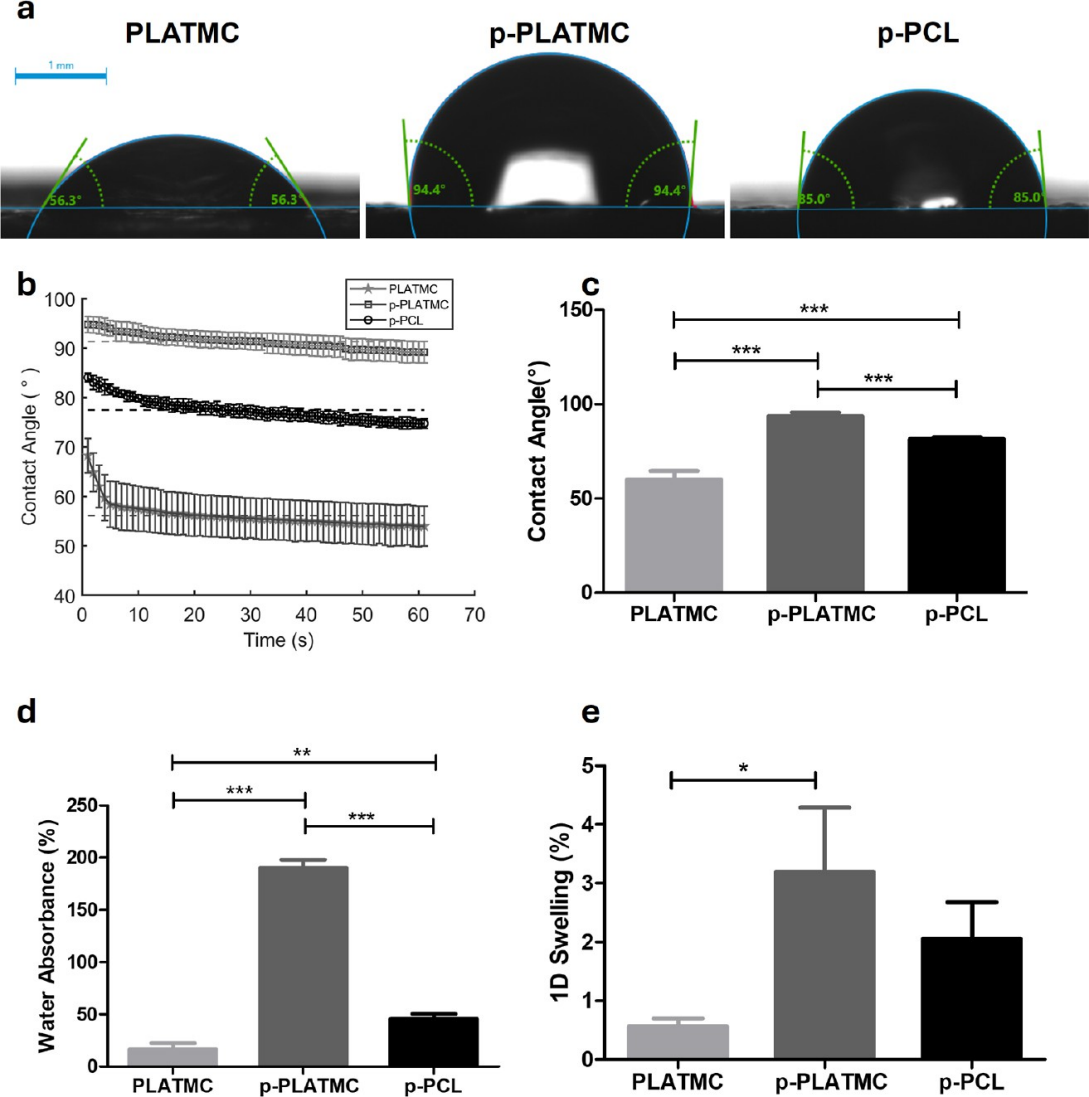
Water contact angle analysis on dry, washed sheet samples during rehydration. (a) Surface-water interaction. (b) Dynamic changes in contact angle over 1 min. (c) Average contact angle within the first 10 s of initial contact. (d) Water absorbance/uptake (%) and (e) the swelling ratio based on 1D length change (%) in 3D-printed rehydrated samples. Asterisks (*) indicate significant statistical differences among the groups (one-way ANOVA), *p < 0.05, **p < 0.01, and ***p < 0.001*.*

**Table 4:** Summary of Scaffold/Sample Properties^a^

	total porosity (%)	modulus of elasticity (*E*) (MPa)	strain (%)	tensile strength (MPa)
PLATMC	27.5 ± 2.5	566 ± 118	178 ± 54	18.54 ± 2.1
p-PLATMC	56.8 ± 3.50	101 ± 20	84 ± 28	4.84 ± 0.6
p-PCL	53.5 ± 1.34	143 ± 2	21.5 ± 2.2	5.4 ± 0.6

a Total porosity shows the contribution of both macro (3D printing porosity of all groups) and microporosity of porous groups.

### Induced Microporosity Affects Material Stiffness and Ductility

The mechanical properties of conventionally printed PLATMC in a dumbbell shape (Figure 7a) were significantly altered by salt leaching printing modification. PLATMC specimens displayed higher ductility, resulting in notably larger strain. Additionally, the nonporous form of PLATMC exhibited a significantly higher (*p* < 0.01) elasticity and tensile strength modulus. Stress–strain curves (Figure 7b) provided insights into the relationship between toughness and the presence of salt-induced porosity in the different specimens: the toughness in PLATMC decreased with NaCl-induced porosity. Microporosity resulted in reduced elongation and tensile strength of PLATMC. Furthermore, introducing internal microporosity significantly reduced the printed specimens’ modulus of elasticity and tensile strength (Figures 7c,d and S2). The results were also compared to the control (p-PCL). Higher molecular weight PLATMC specimens exhibited increased ductility, leading to significantly greater strain than that of p-PCL specimens. The properties of the groups were summarized in Table 4.

**Figure 7:**
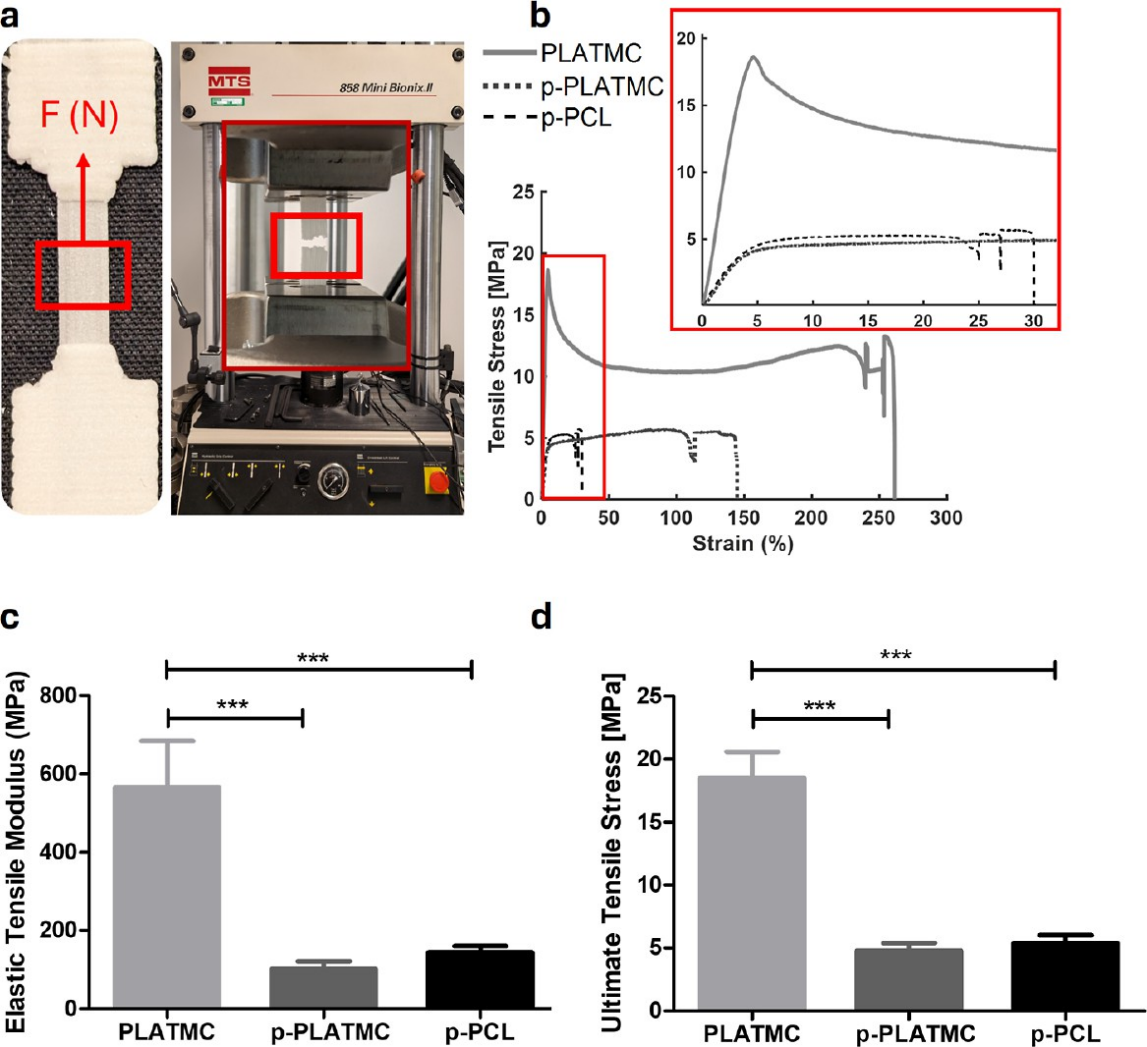
Mechanical tensile test of dog-bone (dumbbell) specimens (*n* = 5). (a) Setup with specimen, (b) stress–strain curve, (c) elastic tensile modulus, and (d) ultimate tensile stress. Asterisks (*) indicate significant statistical differences among the groups (one-way ANOVA), *p < 0.05, **p < 0.01, and ***p < 0.001*.*

### MgCHA Nanoparticles Were Successfully Synthesized

MgCHA nanoparticles were successfully prepared and characterized. MgCHA was found to be round, with particles of almost 80 nm, as obtained from SEM analysis (Figure 8). X-ray diffraction (XRD) confirmed the identity of the HA and the low degree of crystallinity compared to commercial nanohydroxyapatite particles (Sigma-Aldrich/Merck). The ATR-FTIR analysis clearly showed the phosphate bands between 950–1050 cm^–1^ and 550–600 cm^–1^, together with the presence of both the absorbed and occluded water, related to the broad band around 2650–3650 cm^–1^ and the peak at 1660 cm^–1^, respectively. Furthermore, the typical signals of β-carbonation, i.e., the substitution in the phosphate position, were detected, as shown by the CO_3_ stretching signals at 1420 and 1480 cm^–1^ and the bending peak at 870 cm^–1^. Carbonation was successfully observed by TGA analysis, with CO_2_ loss in the range of 600 °C–800 °C, after initial water loss in the range of 25 °C–100 °C. Finally, the effective ionic substitution was confirmed by ICP analysis, where the typical Ca/P ratio of stoichiometric HA of 1.67 was shifted toward 1.85, demonstrating the successful Mg doping and the synergistic interaction of Mg and CO_3_ toward the doping during the synthesis (the table in Figure 8).

**Figure 8:**
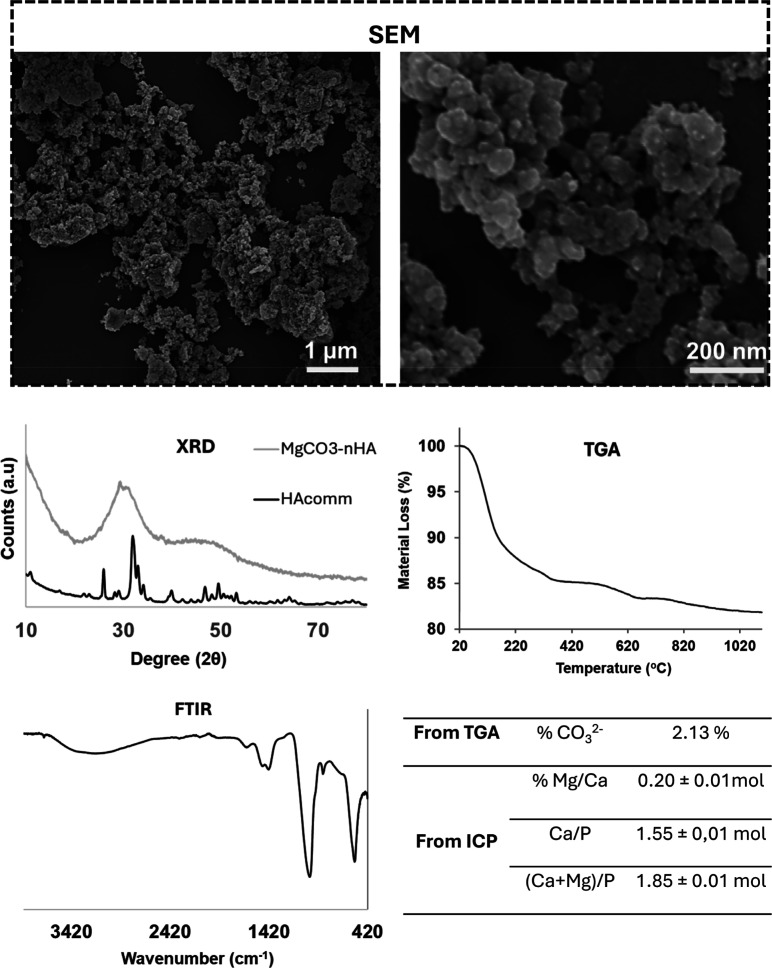
Structural (SEM), crystal (XRD), thermal (TGA), and chemical (FTIR) characterization of synthesized magnesium- and carbonate-doped hydroxyapatite (MgCHA) used as a surface coating on porous PLATMC (p-PLATMC). The SEM images provide particle size, XRD patterns confirm its crystalline phase in comparison to commercial hydroxyapatite (HAcomm), TGA analysis evaluates its thermal stability, and FTIR spectra identify key functional groups, verifying successful doping and synthesis of MgCHA for effective surface modification.

### Dip-Coating of MgCHA on Porous PLATMC

The selection of the liquid for the resuspension solution involved testing two options: DI water^36^ and ethanol,^37,38^ at various compositions. These compositions included 100% DI water, 100% ethanol, and mixtures in intervals of 70–30%, 50–50%, and 30–70% water–ethanol. SEM images revealed that MgCHA particles were distributed more evenly across the scaffold surface when absolute ethanol was present (Figure S3). The concentration of the resuspension solution (% MgCHA in ethanol) was also examined (Figure 9). SEM images depicted cluster formation on scaffolds coated with higher concentrations of the resuspension solution (2.5% and 5%), whereas particles were more uniformly dispersed on scaffolds coated with a 1% MgCHA solution (Figure 9a). However, gravimetric analysis of printed porous scaffolds indicated no significant difference in the coating amount relative to MgCHA concentrations (Figure 9b). Furthermore, micro-CT images revealed that MgCHA particles were found within the porosity of the scaffolds (Figure 9c). Micro-CT scanning also proved that coating was successful, as it depicted a thin layer (white) around the filament due to polymer-ceramics contrast (difference in the X-ray attenuation coefficient).

**Figure 9:**
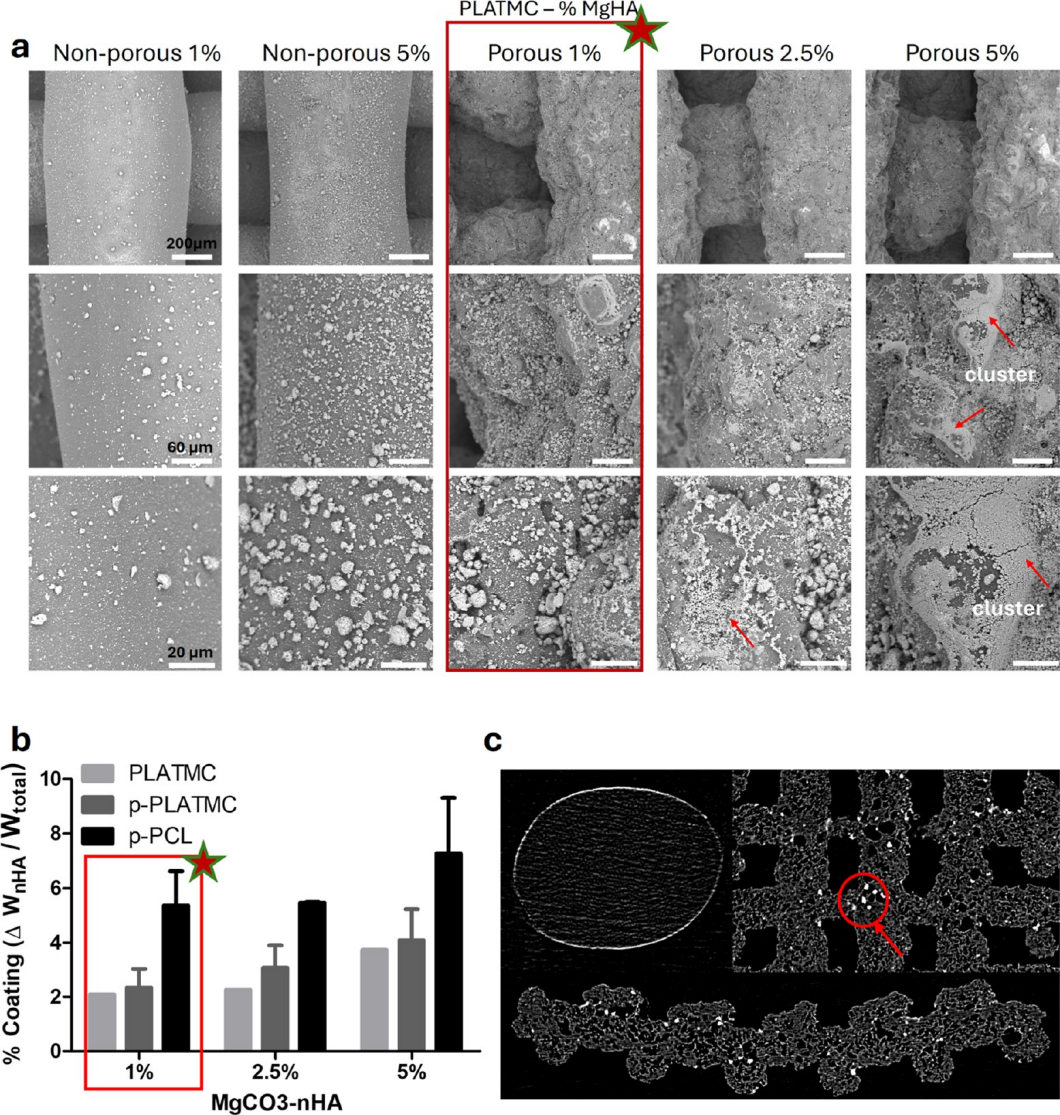
Effect of MgCHA concentration in resuspension on coating efficiency: (a) SEM images showing surface coverage and MgCHA distribution, (b) gravimetric measurement quantifying coating amount on scaffolds with respect to MgCHA content, and (c) micro-CT scanning revealing presence of MgCHA on the surface as well as within the porous structure of printed scaffolds.

### Effect of Microporosity and MgCHA Coating on hBMSC Viability and Proliferation

At all time points and regardless of porosity, most of the hBMSCs were viable on the 3D-printed PLATMC-based scaffolds (Figure 10a). Over time, cells on all scaffold groups elongated, creating a uniformly dispersed network within the scaffold space.

**Figure 10:**
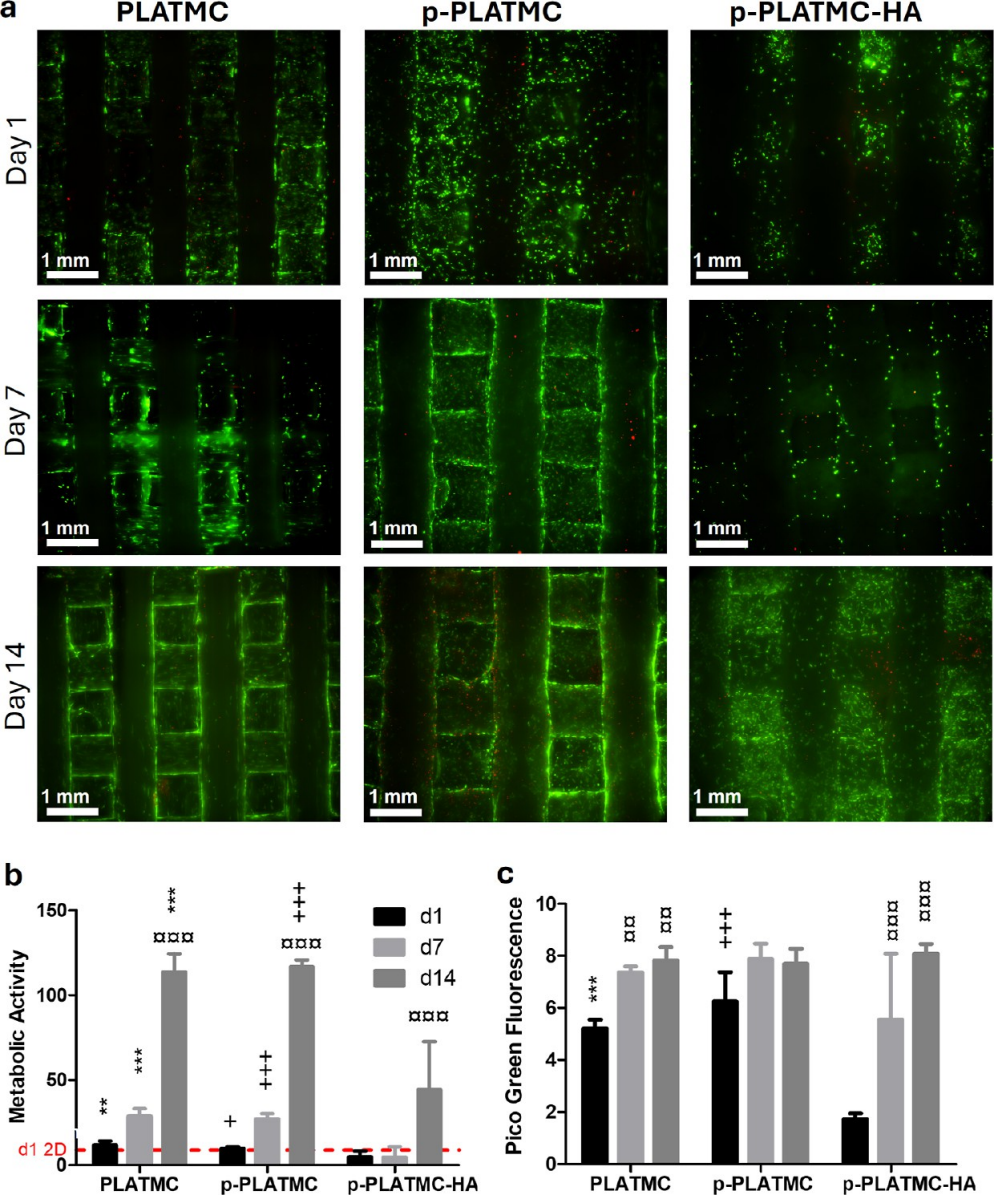
In vitro viability assessment of hBMSC cultured on 3D-printed PLATMC-based scaffolds. (a) Fluorescence live-dead images illustrate the viability of the cells, with live cells indicated in green and dead cells in red; the scale bars are 1 mm. (b) Metabolic activity assessed using the PrestoBlue assay (*n* = 4) and (c) cell proliferation evaluated by the PicoGreen dsDNA assay (*n* = 4). Asterisks (*) denote statistically significant differences between PLATMC and p-PLATMC-HA, plus (+) denotes that between p-PLATMC and p-PLATMC-HA (1-way Anova), while currency signs (¤) indicate differences between time points within the same group (2-way Anova). *, ¤*p* < 0.05; **, ¤¤*p* < 0.01; and ***, ¤¤¤ *p* < 0.001.

Cell viability and activity of the cells were confirmed by measuring the cell metabolic activity (Figure 10b). In contrast, cell proliferation was quantified by PicoGreen dsDNA (Figure 10c). The metabolic activity of the cells increased significantly (*p* < 0.001) from days 1 to 14 across all groups. No significant difference was observed between the nonporous and porous forms of PLATMC. However, they exhibited significantly higher (*p* < 0.05) metabolic activity compared with the coated (p-PLATMC-HA) group at all time points (Figure 10b). Furthermore, the results obtained from dsDNA quantification showed that on day 1, the number of cells was comparable between the nonporous and porous forms of PLATMC groups. Still, those were significantly higher (*p* < 0.01) than those for p-PLATMC-HA (Figure 10c). The number of cells tended to increase across all scaffold groups from day 1 to day 14, indicating the ability of the printed scaffolds to support cell proliferation.

When comparing material chemistry, no significant differences were observed between p-PLATMC and the control (p-PCL) at the early time points on days 1 and 7, indicating that material chemistry had a minimal impact during these periods (Figure 11b). However, on day 14, p-PLATMC demonstrated higher activity compared to p-PCL. Additionally, p-PLATMC and p-PCL effectively supported cell proliferation (as measured by dsDNA content) over time, with a similar performance between the two materials (Figure 11c).

**Figure 11:**
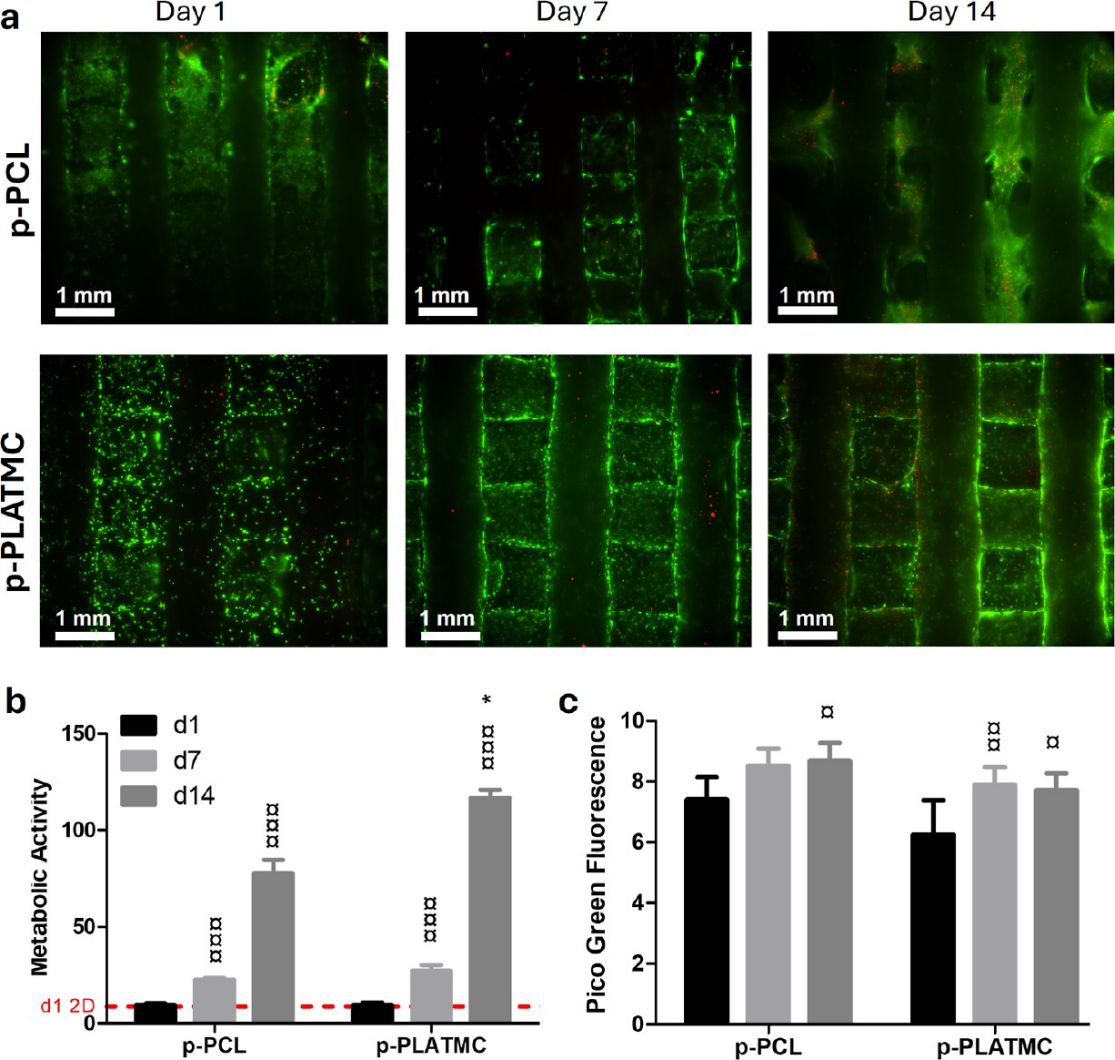
In vitro viability assessment of hBMSC cultured on 3D-printed porous PLATMC scaffolds in comparison with the control (p-PCL). (a) Fluorescence live-dead images illustrate the viability of the cells, with live cells indicated in green and dead cells in red; the scale bars are 1 mm. (b) Metabolic activity assessed using the PrestoBlue assay (*n* = 4) and (c) cell proliferation evaluated by the PicoGreen dsDNA assay (*n* = 4). Asterisks (*) denote statistically significant differences between p-PCL and p-PLATMC, while currency signs (¤) indicate differences between time points within the same group (2-way Anova). *, ¤*p* < 0.05; **, ¤¤*p* < 0.01; and ***, ¤¤¤ *p* < 0.001*.*

### Effect of Microporosity and MgCHA Coating on Osteogenic Potential

Alkaline phosphatase (ALP), an early osteogenic differentiation marker, was quantified after normalization to the total number of cells (Figure 12a). The activity of ALP was significantly elevated from day 7 to day 14 (*p* < 0.001) across all scaffold groups regardless of the presence of microporosity and surface coating. Moreover, it was significantly higher (*p* < 0.005) in porous PLATMC on days 7 and 14 compared with its nonporous form. While porous PLATMC initially had higher activity than the MgCHA-coated group on day 7, the latter escalated rapidly by leveling off the difference by day 14, with no significant difference observed between porous scaffolds and its coated form at the end of the incubation period (Figure 12a). To distinguish between the two microporosity-induced polymeric scaffolds with differing materials/chemistries, porous PLATMC was compared to its counterpart: the control (p-PCL). Furthermore, the control (p-PCL) exhibited a higher ALP activity at each time point (Figure S4).

**Figure 12:**
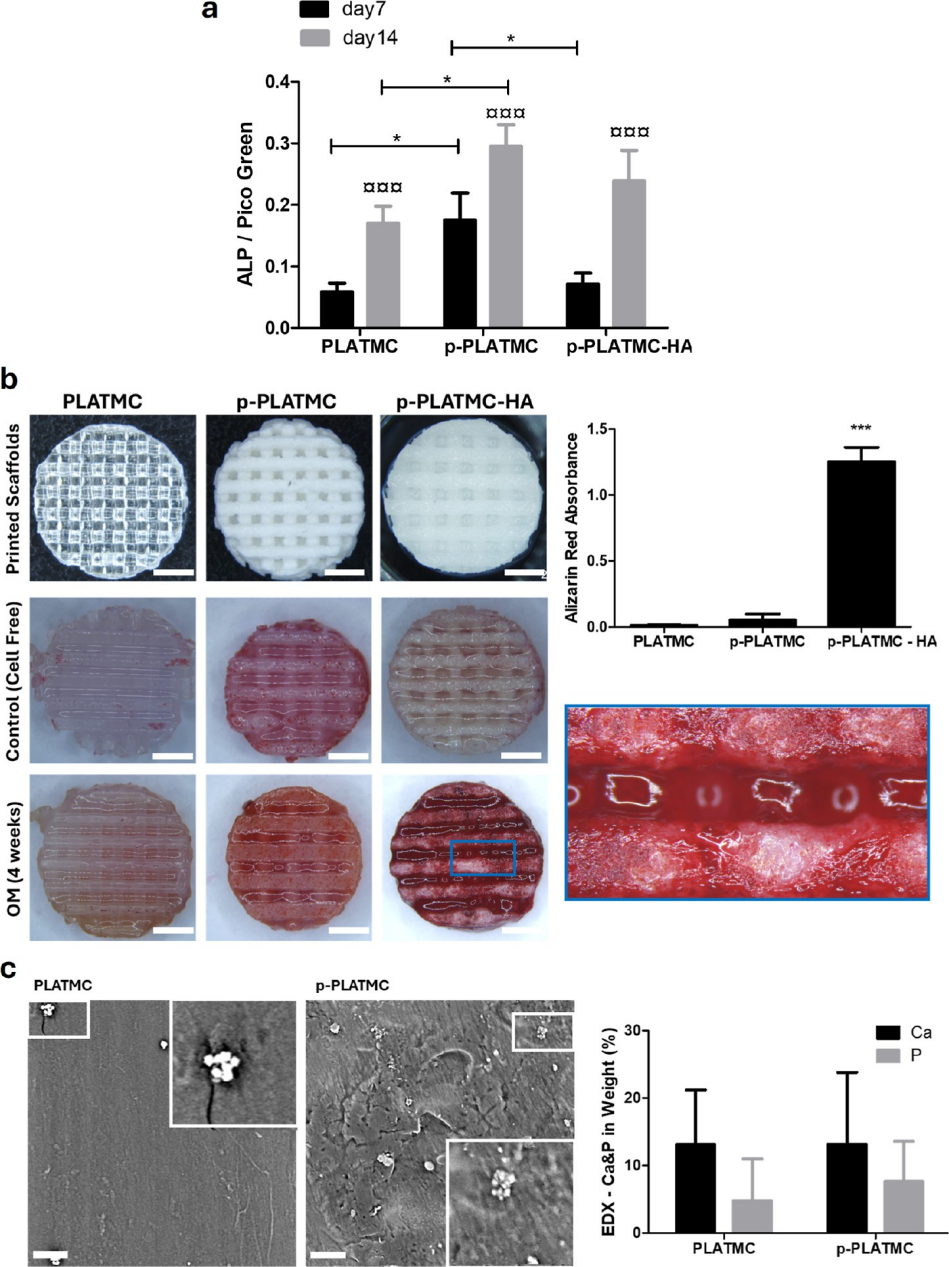
In vitro osteogenic differentiation of hBMSC cultured on 3D-printed scaffolds (a) Alkaline phosphatase (ALP) activity normalized to dsDNA (*n* = 4). (b) Alizarin Red-S staining and absorbance read-out after destaining of cell-free and cell-seeded scaffolds on day 28; the scale bars are 2 mm, (*n* = 3). (c) SEM-EDS images and analysis showing Ca and P formation and deposition, respectively. Asterisks (*) indicate significant statistical differences between the groups (1-way Anova), while currency sign (¤) indicates the difference between time points of the same group (2-way Anova). *, ¤ *p* < 0.05; **, ¤¤ *p* < 0.0; and ***, ¤¤¤ *p* < 0.001*.*

Furthermore, cells cultured on scaffolds were stained with Alizarin Red-S on day 28 of culture to detect calcium deposition (Figure 12c). Compared with cell-free scaffolds, all scaffolds seeded with cells displayed a color change and contrast. P-PLATMC-HA exhibited significantly higher mineral deposition (*p* < 0.001) than other groups. Both porous and nonporous forms of PLATMC scaffold groups disclosed Ca–P mineralization as shown by the SEM-EDS imaging and analysis (Figure 12c) with no significant differences.

Immunofluorescence analysis in the Supporting Information (Figure S5) showed that all scaffold groups, PLATMC, porous PLATMC, and HA-coated porous PLATMC supported the expression of RUNX2 and Collagen-I. Among them, porous PLATMC demonstrated notably stronger RUNX2 fluorescence signals and higher Collagen-I expression levels than the nonporous PLATMC scaffolds. These findings suggest that introducing filament-level microporosity may promote the osteogenic differentiation of seeded cells.

### Release of Ions during Cell Culture in Osteogenic Medium

The concentrations of calcium (Ca^2+^), phosphate (PO_4_^3–^), and magnesium (Mg^2+^) ions in the MgCHA samples were significantly higher (*p* < 0.05) at both time points compared to the control (osteogenic medium). However, the concentration of Ca ions in both the control and MgCHA sample media significantly decreased (*p* < 0.001). In contrast, the concentration of PO_4_^3–^ increased significantly (*p* < 0.001), and that of Mg^2+^ remained relatively unchanged from day 1 to day 28, especially in the MgCHA (target) group (Figure 13). The quantification of ions released into the cell culture medium is detailed in Table 5. Specifically, on day 1 of the ICP sample analysis, the data showed that Ca^2+^ concentration in the MgCHA group was 1800 mg/L, while in the control medium, it was 1440 mg/L. This represents a net increase of 360 mg/L of Ca^2+^ attributed to the release from the MgCHA coating. Based on the stoichiometric calcium-to-hydroxyapatite (HA) ratio of 10:1 (eq 7), this corresponds to the release of approximately 36 mg/L of HA. Similarly, PO_4_^3–^ concentrations were 1000 mg/L in the MgCHA group and 800 mg/L in the control, indicating a difference of 200 mg/L attributable to the scaffold. Theoretically, the initial MgCHA coating applied to each scaffold was estimated to contain 600 mg of the material, with target ion release values of 6000 mg/L for calcium and 3600 mg/L for phosphate. From the observed release, it is estimated that approximately 6% of MgCHA coating was released into the medium on day 1. A similar ion release trend was observed on day 28, suggesting a relatively sustained and controlled dissolution behavior of the MgCHA coating over time.

7 $$Mg{CO}_{3\left(dope\right)}-{Ca}_{10}{\left({PO}_{4}\right)}_{6}{\left(OH\right)}_{2}⇌10{Ca}^{2+}+6{\left({PO}_{4}\right)}^{3-}+2{OH}^{-}+{Mg}^{2+}$$

**Figure 13:**
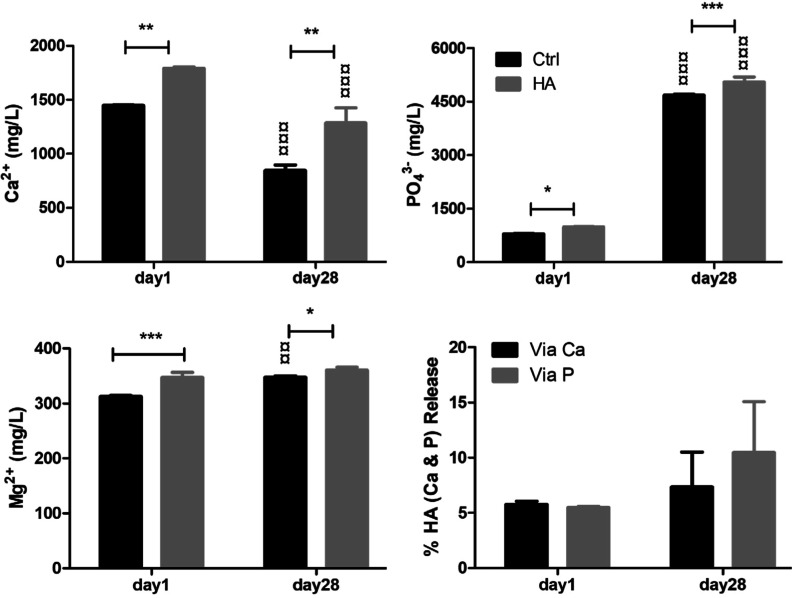
ICP-OES (inductively coupled plasma optical emission spectrometry) analysis of calcium, phosphate, and magnesium ions present in cell culture media collected from the control group (p-PLATMC) and the target group (p-PLATMC-HA) at the initial and final time points. Osteogenic supplemented media of samples in the cell culture collected in Eppendorf tubes (about 500 μL). Samples were diluted by 20 times for ICP-OES. Asterisks (*) indicate significant statistical differences between the group (p-PLATMC) and the target group (p-PLATMC-HA) at the same time point, while currency sign (¤) indicates the difference between time points of the same group (*n* = 3). *, ¤*p* < 0.05; **, ¤¤*p* < 0.01; and ***, ¤¤¤ *p* < 0.001*.*

**Table 5:** Summary of ICP-Based Ion Concentrations Released from MgCHA Samples in Cell Culture on Day 1

ICP samples day 1 (Avg)	Ca^2+^ mg/L	(PO_4_)^3–^ mg/L	HA coating ${Ca}_{10}{\left({PO}_{4}\right)}_{6}{\left(OH\right)}_{2}⇌$ mg/L/sample	10Ca^2+^ mg/L	6(PO_4_)^3–^ mg/L
+ HA (target)	1800	1000	600 (HA on a sample)	6000	3600
– media (ctrl)	1440	800			
= difference	360	200	36 (HA released)	360	200
toxicity value^39^			500	5000	3000
% HA released			100 × (36/600) = 6	6	6
toxicity comparison			36 ≪ 500		

## Discussion

Although extrusion-based 3D printing enables the creation of scaffolds with considerable porosity (in the millimeter range) by adjusting the spacing between printed filaments, the filaments themselves are typically nonporous and possess a smooth surface.^40^ The present study aimed to introduce internal microporosity on printed filaments of p-PLATMC scaffolds coated with magnesium-carbonate-doped hydroxyapatite (MgCHA) and evaluate their osteogenic potential in vitro. The study also evaluated the effects of these scaffolds on the viability, proliferation, and osteogenic differentiation of human bone marrow-derived mesenchymal stromal cells (hBMSCs).^23^

### Thermal Properties of the Developed Granules

PLATMC is a block copolymer consisting of PLA and TMC. The low-temperature endothermic peak observed in DSC is most likely associated with the glass transition temperature (*T*_g_) of PLA, typically reported between 55 and 65 °C. This peak may also reflect the melting of small crystalline regions or disordered polymer segments, potentially corresponding to TMC-rich domains known for their limited crystallization capacity. The high-temperature endothermic peak is characteristic of the melting temperature (T_m_) of PLA domain within the PLATMC copolymer. Previous studies support this interpretation: Jain et al. reported a melting temperature of 158 °C for PLATMC,^33^ while Ji et al. observed that increasing TMC content leads to a decrease in *T*_g_, consistent with the presence of more flexible, amorphous TMC segments.^41^ In contrast, PCL exhibited a distinct melting point at approximately 71 °C, and its thermal degradation occurred between 394 and 433 °C.^42^ It has also been reported that NaCl reduces the melting temperature of polymers by interfering with crystalline domain formation.^43^ In the present study, the incorporation of NaCl into PLATMC resulted in a decrease in the melting temperature and a decrease in the intensity and enthalpy of both endothermic peaks, indicating a reduction in overall crystallinity. This can be attributed to the disruption of polymer chain packing and molecular organization due to the presence of salt particles. Moreover, variations in the melting temperatures observed across the polymer samples may also reflect molecular weight and composition differences, which influence thermal transitions and crystallization behavior. While PLATMC has a molecular weight around 100 kDa, as indicated by size exclusion chromatography (SEC) in the study by Jain et al.,^33^ PCL has an average molecular weight of 45 kDa. We observed that PLATMC groups had lower decomposition temperatures and thermally degraded faster than the control (PCL) group. In fact, PLA in general has a faster degradation rate when compared to PCL, and the relative proportions in blends can be used to control the degradation time.^44^ The copolymer PLATMC, composed of PLA and TMC, undergoes thermal degradation at a lower temperature than PCL. This is likely due to both of its components, particularly TMC, having higher thermal degradation rates than PCL, which leads to the copolymer decomposing at a lower temperature.^6^ Higher residuals were observed when air was used, likely due to the reaction of NaCl with oxygen, forming NaClO_3_.

### Extrusion Dynamics and Shear-Dependent Behavior of Polymers: Rheology and Simulation Prediction

All developed granules exhibited typical shear-thinning behavior characterized by a decrease in viscosity with increasing shear stress. Adding NaCl to PLATMC increased the zero-shear viscosity but reduced viscosity at elevated shear rates. This behavior can be attributed to the alignment or redistribution of NaCl particles under high pressures, which reduces resistance to flow by disrupting the polymer chains through a disentangling phenomenon.^45^ In certain cases, the presence of these particles can create more uniform paths for the polymer, further lowering the viscosity and ultimately increasing the printing speed in PLATMC-NaCl systems. Additionally, the polymer weight fraction in composite granules is half (50%) of that of PLATMC granules alone, which may also influence the polymer flow behavior.

The primary goal of COMSOL modeling was to investigate the relationship between printing pressure and printing speed during the extrusion process, specifically how printing pressure (in bars) affects the velocity of the melted polymer as it passes through the nozzle. In addition to analyzing real-time behavior, the simulation provided preliminary estimates of the maximum attainable printing speeds, offering valuable predictive insights before experimental validation. Researchers have made an effort to investigate the complex viscosity using the Carreau model. Previous studies have demonstrated the effectiveness of the Carreau model in analyzing the complex viscosity of polymers and polymer-based composites and in characterizing the rheology of polymeric and composite systems.^46,47^ Compared to the simpler power law model, the Carreau model offered a more robust fit, particularly at higher shear rates, which is typical during extrusion through narrow nozzles under elevated pressures. This allowed for a more realistic simulation of extrusion conditions encountered during high-speed 3D printing, making the model an effective tool for predicting extrusion behavior and optimizing print parameters.

The Hagen–Poiseuille and flow rate equations state that, for a Newtonian fluid, the velocity is directly proportional to the square of the nozzle diameter (2*r*) and inversely proportional to the nozzle length (E4). It is important to note that although polymeric materials do not behave as Newtonian fluids in real-world applications, using the Newtonian equation can still be valuable. It provides a basic framework for understanding the relationship between key parameters, offering simplified insights into the material’s behavior under specific conditions. Based on the computational analysis and the Hagen–Poiseuille relation, polymer flow velocity is expected to increase with a larger nozzle diameter and shorter nozzle length. The printing speed of PLATMC was reported to be between 2 and 5 mm/s at 195 °C and 8 bar, following a 15 min preheating at 220 °C using a 0.4 mm nozzle diameter.^5^ This finding is consistent with the computational estimates from our study. However, a printing speed of 2 mm/s is relatively slow at the beginning, as the speed generally increases over time due to higher molecular weight degradation caused by increased thermo-oxidative degradation of PLATMC.^33^ Our rheology data and simulations, based on a 30 min preheating at 200 °C, represent an early printing window when degradation is minimal. Increasing the nozzle diameter could potentially enhance the relatively slow printing speed of 2 mm/s with a 0.4 mm nozzle. Additionally, incorporating NaCl into the polymer increases the zero-shear/initial viscosity, as both rheological data and experimental results show. The presence of 40–90 μm particles may have contributed to clogging due to agglomeration and uneven heat distribution compared to pure PLATMC. For convenience in this study, a 0.6 mm nozzle diameter was selected. This nozzle size enabled printing tall and large structures with high shape fidelity and quality. Integrating rheology, mathematical modeling, and simulation provided valuable insights into predicting and understanding polymer flow during extrusion-based printing. It also facilitated the optimization of key parameters, including nozzle type, diameter, and length as well as printing pressure, speed, and their associated limitations and allowances.

### Effect of Filament Porosity on Surface and Bulk Properties of the Scaffolds

It is well established that the initial salt concentration affects the overall porosity.^16^ Introducing microporosity to the printed filaments is crucial not only for guiding cell behavior but also for tailoring the surface roughness and bulk stiffness of the scaffolds.^16^ The degradation rate can also be influenced by this microporosity.^17^ Additionally, microporosity can enhance the scaffolds’ swelling properties (water uptake), which is vital for nutrient delivery to cells.^15^ Supporting this, the findings in this study also confirmed that NaCl leaching directly impacted material properties, such as surface roughness, by altering the porosity. Salt leaching and subsequent microporosity led to a significant change in the microstructure of PLATMC scaffolds, with notable differences observed in the surface and cross-sectional morphology between the nonporous and porous form of PLATMC. However, since the initial NaCl content was the same, no significant differences were observed in overall porosity and pore size between p-PLATMC and the control (p-PCL). Nevertheless, the higher surface roughness observed in p-PLATMC compared to the control (p-PCL) may be attributed to the fact that these are fundamentally different materials, each distinctly possessing unique surface tension and surface chemistry characteristics. Variations in surface roughness could influence the interaction between water and the material upon initial contact. Typically, for the same type of material, a rougher surface tends to exhibit greater hydrophobicity because it impedes water interaction.^48,49^ Although PLATMC appears more hydrophilic (with a lower contact angle) than its porous counterpart (p-PLATMC), the increased surface area due to the microporosity in p-PLATMC resulted in a much higher water absorption capacity (*p* < 0.01) compared to PLATMC once the scaffolds were fully saturated. Initially, microporosity played a dominant role in water absorption, until the scaffolds reached saturation. After this point, the hydrophilic nature of the material further contributed to the increased water absorption in the p-PLATMC.

In contrast, when comparing p-PLATMC to the control (p-PCL) group, the porosity was no longer a distinguishing factor. Although p-PCL appeared slightly more hydrophilic than p-PLATMC based on contact angle measurements, p-PLATMC absorbed more water due to the inherently more hydrophilic nature of PLATMC. Additionally, although the study by Hassan et al.^5^ did not incorporate salt leaching or microporosity, their findings showed that the wettability of PLATMC was higher than that of PCL, with a lower contact angle for both 3D-printed and cast sheet forms, like our results. This clearly demonstrates that both microporosity and the type of material used significantly influence the water absorption and swelling characteristics.

3D printing of polymeric scaffolds at high temperatures without any modification typically results in rigid scaffolds.^40^ It is well established that porosity introduces voids or pores within the material, which act as sites for stress concentration. These pores can initiate microcracks and accentuate failure under loading, as stress is concentrated around them, reducing the material’s ability to withstand tensile, compressive, and flexural stresses.^50^ Our previous research employed the nonsolvent induced phase separation (NIPS) method to create more stretchable scaffolds, benefiting from the high porosity and elongated polymer chains in solution-based printing.^23,51^ The characteristics of the copolymer PLATMC make it a strong alternative to NIPS scaffolds due to its inherent elongation properties and higher strength, which arise from its longer polymer chains, high molecular weight, and specific composition. PLATMC has a more amorphous structure due to the incorporation of trimethylene carbonate (TMC) units, which disrupt the regular crystalline packing of poly­(l-lactide) (PLLA) chains, leading to greater chain mobility.^52^ In contrast, PCL is a semicrystalline polymer with tightly packed crystalline regions, which restrict chain movement and reduce flexibility.^53^ Jain et al. also demonstrated that PLATMC has a molecular weight exceeding 100 kDa and consists of 60 mol % l-lactide and 40 mol % trimethylene carbonates (TMC).^33^ As a result, PLATMC exhibited higher tensile strength, Young’s modulus, and strain compared to p-PLATMC and the control (p-PCL). However, when comparing p-PLATMC and the control, both show similar strengths, but p-PLATMC demonstrates greater elongation due to its inherent properties. The higher ductility of PLATMC makes it ideal for applications that require stretchability.

### Effect of Microporosity-Regulated Roughness on Scaffold-hBMSC Interaction

Porosity is a critical factor in bone formation, both in vitro and in vivo.^10,14,54^ This study hypothesized that the micropores induced by the salt leaching method (40–90 μm) could enhance the performance of hBMSC. As discussed previously, microporosity influences both surface roughness and bulk stiffness. Surface roughness plays a crucial role in facilitating cell adhesion on scaffolds, with higher surface roughness enhancing initial cell adhesion and supporting cell growth.^55^ Moreover, changing surface roughness has been found to determine the ideal roughness for enhancing the osteogenic differentiation of hBMSC.^56,57^ In line with these studies, introducing microporosity in this work enhanced surface roughness and surface area, allowing for greater cell attachment and promoting a higher proliferation. Additionally, it has been reported that stiff substrates encourage cells to develop cytoskeletal stress fibers, which in turn promote enhanced cell spreading and proliferation.^58–60^ Osteogenic differentiation has also been shown to be greater on stiffer substrates.^61,62^ While introducing microporosity in this study reduced the material’s strength and stiffness, it simultaneously enhanced surface roughness, which positively influenced cell activity and differentiation. Our findings suggest that the balance between stiffness and roughness remains within a range that supports both cell proliferation and osteogenic differentiation. Nevertheless, the literature presents challenges in determining the optimal values for total porosity, pore size, stiffness, and roughness that promote hBMSC proliferation and osteogenic differentiation. These challenges may stem from variability in scaffold materials, shapes, surface modifications, fabrication methods, and culture conditions across different studies.^10,63^

### Effect of MgCHA Coating on Osteogenic Potential of hBMSC

In this study, inorganic MgCHA nanoparticles were developed through a neutralization process involving Mg^2+^ ions to mimic the natural microenvironment of bone mineral formation and growth. This method imparts biomimetic and biodegradability properties to the hydroxyapatites, resulting in higher bioactivity than stoichiometric hydroxyapatite. The bioactivity of hydroxyapatite under physiological conditions is significantly influenced by two main factors: the degree of crystallinity and the distortion of its crystal lattice due to the incorporation of foreign doping elements, which can be utilized to make the material more comparable to natural bone tissue.^64^ Specifically, doping with ions such as Mg and CO_3_ allows for a chemically enhanced inorganic phase resembling natural hydroxyapatite while also reducing the crystallinity of the HA, which is further decreased when synthesis occurs at mild temperatures (e.g., 25 °C). This study selected PLATMC due to its promising osteoconductive potential and application.^5,65,66^ Magnesium-doped hydroxyapatite was selected as the osteoconductive coating material due to the well-documented role of magnesium ions (Mg^2+^) in promoting osteogenic differentiation. Several studies have demonstrated that Mg^2+^ can upregulate key osteogenic markers, including RUNX2 and Collagen-I (type 1), which are critical for early and late stages of osteoblast differentiation.^67^ The underlying mechanism is thought to involve the activation of the integrin–focal adhesion kinase (FAK)–extracellular signal-regulated kinase (ERK) signaling pathway, which plays a pivotal role in transducing extracellular matrix signals into intracellular osteogenic responses.^68^ By incorporating Mg^2+^ into the hydroxyapatite lattice, the coating not only mimics the mineral component of native bone but also contributes to the biochemical stimulation of stem cell differentiation, enhancing the scaffold’s overall bone-regenerative potential.

Therefore, the porous form (p-PLATMC) was dip-coated with MgCHA at a concentration of 1% (0.1 g in 10 mL = 10,000 mg/L) for surface modification and activation. Approximately 2% of the dry weight of the scaffolds consisted of MgCHA (around 0.6 mg/mL = 600 mg/L). The coating covered a significant area of the scaffold surface, ensuring adequate exposure for the cell interaction. Initial live/dead results (Figure 10) appeared to show a lower number of adherent cells on the MgCHA-coated scaffolds (p-PLATMC-HA) at days 1 and 7, which might superficially suggest cytotoxic effects. However, this observation did not correspond to the actual cytotoxicity. Most of the cells present at these early time points stained green, indicating that they remained viable despite reduced initial adhesion. By day 14, viable cells fully colonized the scaffold surfaces, reflecting improved cell proliferation and viability over time. These findings were corroborated by PrestoBlue metabolic activity assays and DNA quantification via PicoGreen, both of which showed increased cell numbers over the 14 day culture period. Although MgCHA did not significantly enhance cell proliferation or osteogenic differentiation at early time points as initially expected, ALP activity increased significantly (*p* < 0.001) from day 7 to day 14 with no significant differences from other groups at day 14. Notably, the ALP activity ratio between days 7 and 14 was 1.7 for porous PLATMC and 3.3 for the MgCHA-coated form, indicating a greater increase in activity. The mineralization exhibited in the MgCHA group was higher than in the other groups in this study, contrasting with the findings of Hassan et al., who reported no significant differences in the osteogenic differentiation of hBMSC on PLATMC/HA blend groups.^31^ In their study, the PLATMC/HA blend groups, scaffolds with 10% HA exhibited similar osteogenic behavior to those made from PLATMC alone.^31^ The difference in outcome between these studies could stem from the application of doped HA as a surface coating in this study rather than blending regular HA into the scaffold. This distinction can affect factors such as the HA distribution, scaffold surface properties, and release profile of bioactive ions, all of which are important for optimizing bone tissue engineering applications.

Ion release is an important aspect of scaffold performance in bone tissue engineering. Some of the observations from this study may be explained by the gradual increase in cytotoxicity observed at calcium concentrations ranging from 50 to 500 mg/L.^39^ Hydroxyapatite nanoparticles at high concentrations (>500 mg/L) can cause cytotoxic effects, while lower concentrations (below 500 mg/L) could promote better cell attachment, proliferation, and differentiation. Toxicity reference set calcium and phosphate limits at 5000 mg/L and 3000 mg/L, respectively, with an HA release threshold of 500 mg/L^39^ determined by the stoichiometric ratio. The release observed (36 mg/L) fell significantly below toxic levels, indicating that the MgCHA samples were unlikely to be toxic. This suggests a controlled and gradual release of MgCHA from the scaffolds, well below the toxicity threshold (500 mg/L), indicating that the coating is unlikely to induce cytotoxic effects due to the slow and continuous release of bioactive ions (Ca and P).

Alpha-MEM media contain calcium (Ca^2+^), phosphate (PO_4_^3–^), and magnesium (Mg^2+^), whereas DPBS, used for washing, lacks Ca^2+^ and Mg^2+^ but is rich in phosphate salts such as KH_2_PO_4_ and Na_2_HPO_4_. As a result, phosphate (PO_4_^3–^) levels were higher than those of Ca^2+^ and Mg^2+^ due to the influence of DPBS after day 1. Phosphorus levels continued to rise from day 1 to day 28, possibly due to the cellular secretion of P, which accumulates in the media. Although both Ca^2+^ and P are vital for cell functions, phosphorus is more prevalent inside cells and transitions more freely between cellular compartments than the tightly regulated calcium.^69^ Detecting phosphorus via EDX was more difficult, showing weaker signals than those of calcium. The observed decrease in Ca^2+^ from day 1 to 28 may reflect its dynamic cellular regulation,^70^ as it plays a key role in signaling pathways. Elevated Mg levels in MgCHA scaffolds are attributed to MgCO_3_ doping, though the effect is modest due to the low doping level, with Ca and P remaining the dominant elements in hydroxyapatite.

## Conclusions

This study aimed to address the limitations of fabricating PLATMC-HA composites, including the nonporous nature of printed filaments and the use of physically blended stoichiometric hydroxyapatite (HA). A novel approach was taken by integrating NaCl leaching with 3D printing to produce porous and rough PLATMC filaments. Combining these processes resulted in scaffolds with macroporosity from 3D printing and uniform microporosity from salt leaching, which significantly enhanced their structural, surface, and water absorption properties. Introducing microporosity through salt leaching altered the material’s roughness, stiffness, and mechanical properties, resulting in scaffolds that were more favorable for cell proliferation and osteogenic differentiation.

The unique mechanophysical properties of the porous scaffolds were directly linked to their ability to support the proliferation and osteogenic differentiation of hBMSC. All scaffold groups demonstrated the potential for mineralization, as indicated by ALP activity and Alizarin Red-S staining. Notably, the MgCHA-coated scaffolds showed higher mineralization, further supporting their osteoconductive properties. The significant changes in calcium, phosphorus, and magnesium concentrations in the MgCHA-coated samples compared to the control groups suggest that MgCHA may create an environment that enhances matrix mineralization. While releasing ions such as calcium, phosphorus, and magnesium from scaffolds might raise concerns about potential adverse effects on cell behavior and overall scaffold performance, the concentrations observed in this study were significantly lower than the toxicity threshold reported in the literature. These findings indicate that the ion concentrations used in this study are safe and minimize the risk of negative impacts on the cell viability and scaffold functionality. Overall, integrating NaCl leaching and MgCHA coating on PLATMC scaffolds provides a promising approach to tailor surface and bulk properties and enhances the osteogenic potential of 3D-printed scaffolds for bone tissue engineering applications.

## Supplementary Material



## Notes

Additional COMSOL simulation, mechanical testing, MgCHA coating optimization, ALP activity, immunofluorescence images showing RUNX2 and COL-I expression, and calculations and steps of NaCl-based porosity in 3D-printed scaffolds (PDF)
