# Supplementary material for: Osteogenic Potential of 3D-Printed Porous Poly(lactide-co-trimethylene carbonate) Scaffolds Coated with Mg-Doped Hydroxyapatite
Source: ACS Appl Mater Interfaces. 2025 May 15;17(21):31411–33. doi: 10.1021/acsami.5c03945 (PMC12123626; doi:10.1021/acsami.5c03945)
Supplement: Supplementary file 1 [file am5c03945_si_001.pdf]

Supporting Information

Osteogenic Potential of 3D-Printed Porous Poly(Lactide-co-Trimethylene Carbonate) Scaffolds Coated with Mg-Doped Hydroxyapatite

Mehmet Serhat Aydin[1], Carmen-Valentina Nicolae[2], Elisabetta Campodoni[3], Samih Mohamed-Ahmed[1], Masoumeh Jahani Kadousaraei[1], Mohammed Ahmed Yassin[1], Cecilie Gjerde[1], Monica Sandri[3], Izabela-Cristina Stancu[4], Ahmad Rashad[1,5*], Kamal Mustafa[1*]

[1]Center of Translational Oral Research (TOR), Department of Clinical Dentistry, University of Bergen, 5009, Bergen, Norway.

[2]Advanced Polymer Materials Group, Faculty of Chemical Engineering and Biotechnologies, National University of Science and Technology Politehnica Bucharest, Bucharest 011061, Romania.

[3]Institute of Science, Technology and Sustainability for Ceramics (ISSMC-CNR), Faenza RA, 48018, Italy

[4]Faculty of Medical Engineering, National University of Science and Technology Politehnica Bucharest, Bucharest 011061, Romania.

[5]Bioengineering Graduate Program, Aerospace and Mechanical Engineering, University of Notre Dame, Notre Dame, Indiana, 46556, United States.

[*]Authors to whom any correspondence should be addressed.
Email: ahmad.elsebahy@uib.no and kamal.mustafa@uib.no

Keywords: Polymers, doped-Hydroxyapatite, Salt Leaching, Microporosity, Bone Tissue Engineering.

Results

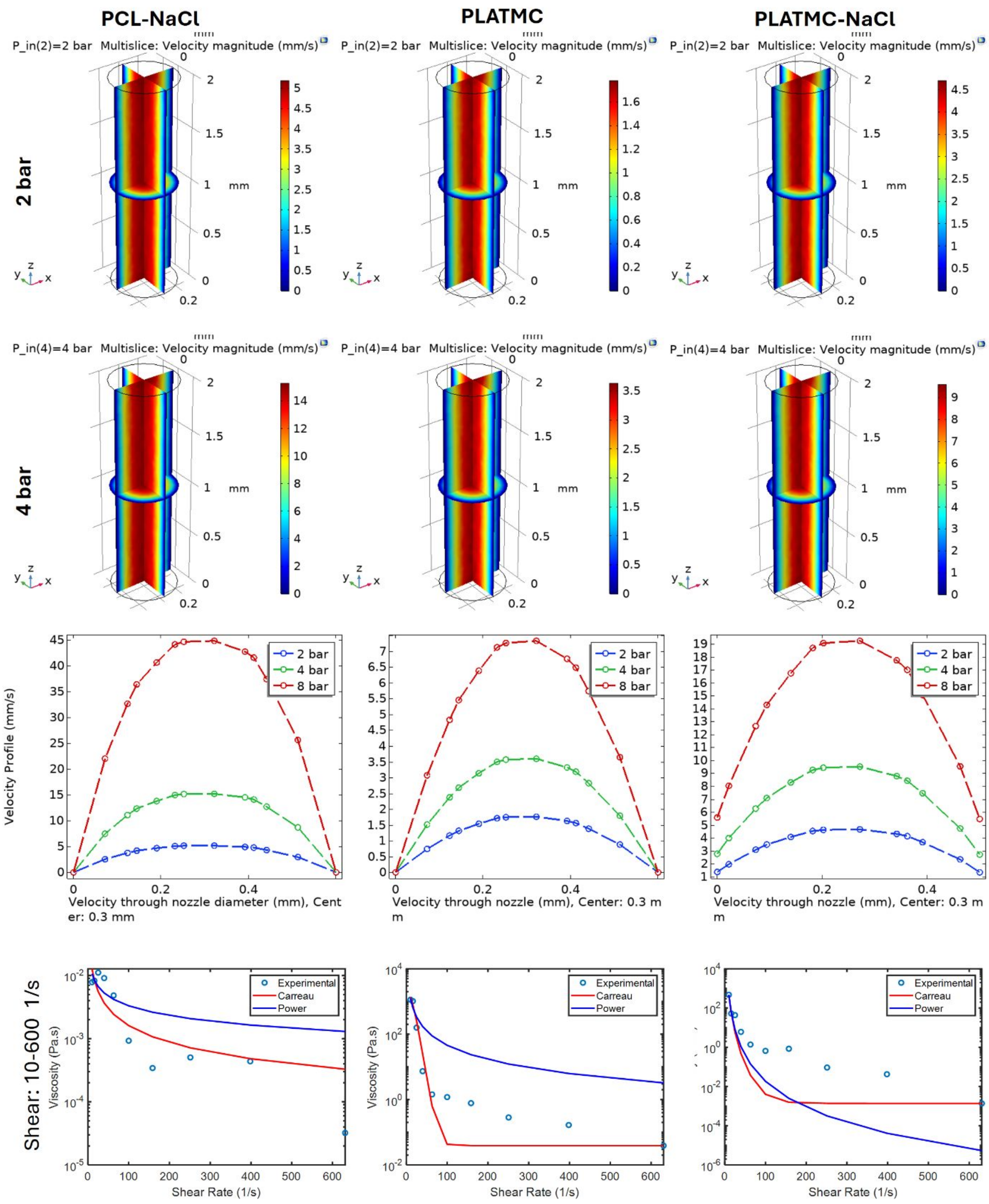

PCL-NaCl
PLATMC
PLATMC-NaCl
2 bar
4 bar
P_in(2)=2 bar Multislice: Velocity magnitude (mm/s)
P_in(4)=4 bar Multislice: Velocity magnitude (mm/s)
mm
Velocity Profile (mm/s)
2 bar
4 bar
8 bar
Velocity through nozzle diameter (mm), Center: 0.3 mm
Velocity through nozzle (mm), Center: 0.3 mm
Shear: 10-600 1/s
Viscosity (Pa.s)
Experimental
Carreau
Power
Shear Rate (1/s)

Figure S1. COMSOL simulation and mathematical modeling curve fitting based on rheological data. Multislice color heatmap of velocity magnitude (mm/s) as shown in at inlet pressure of 2 and 4 bars. Velocity profile through nozzle diameter of 0.6 mm at 2, 4, 8 bars. Carreau and power model curve fitting vs experimental data showing viscosity (Pa.s) vs shear rate (10-600 1/s).

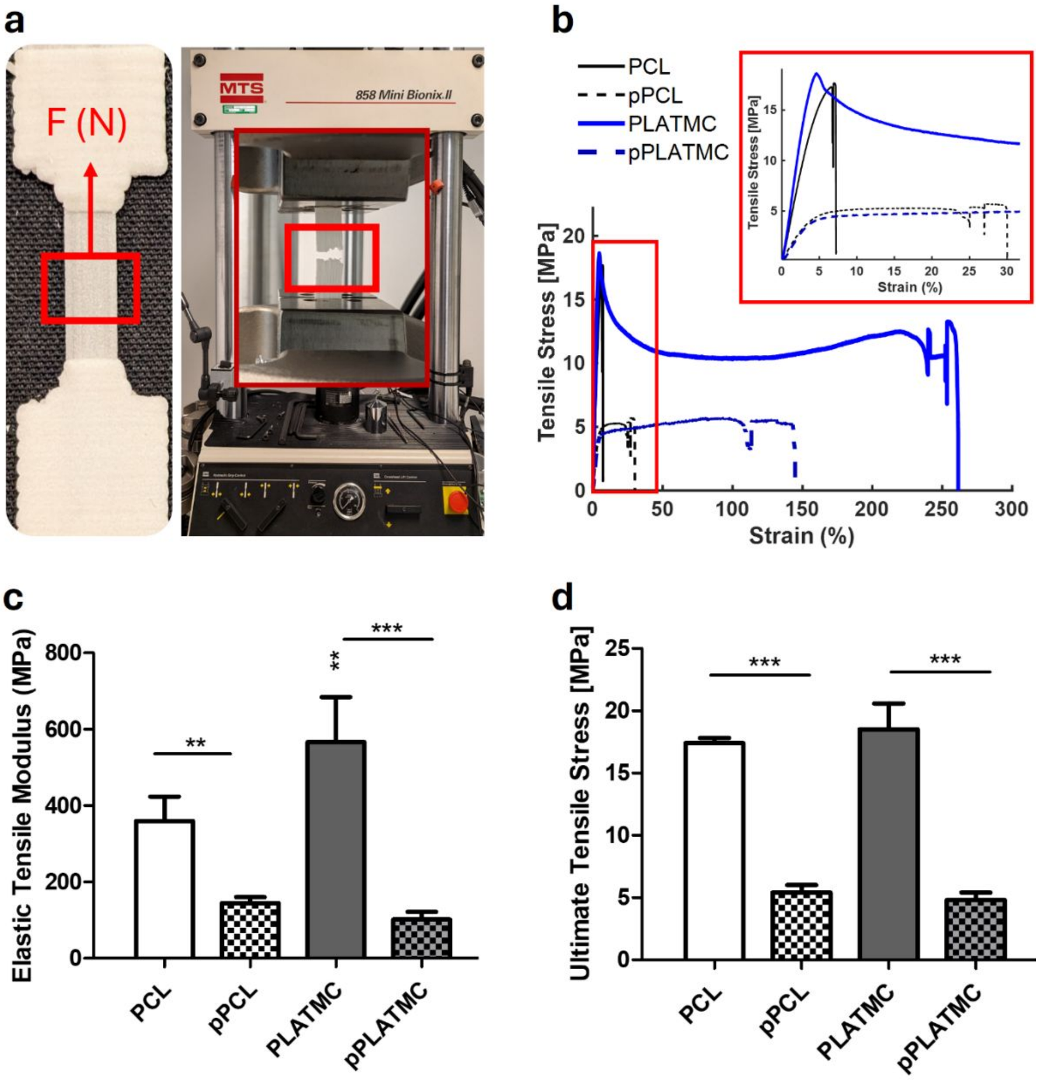

Figure S2. Mechanical tensile test of dog-bone (dumbbell) specimens **(Including Non-porous PCL). (a)** Setup with specimen, **(b)** Stress-strain curve, **(c)** elastic tensile modulus, **(d)** ultimate tensile stress.

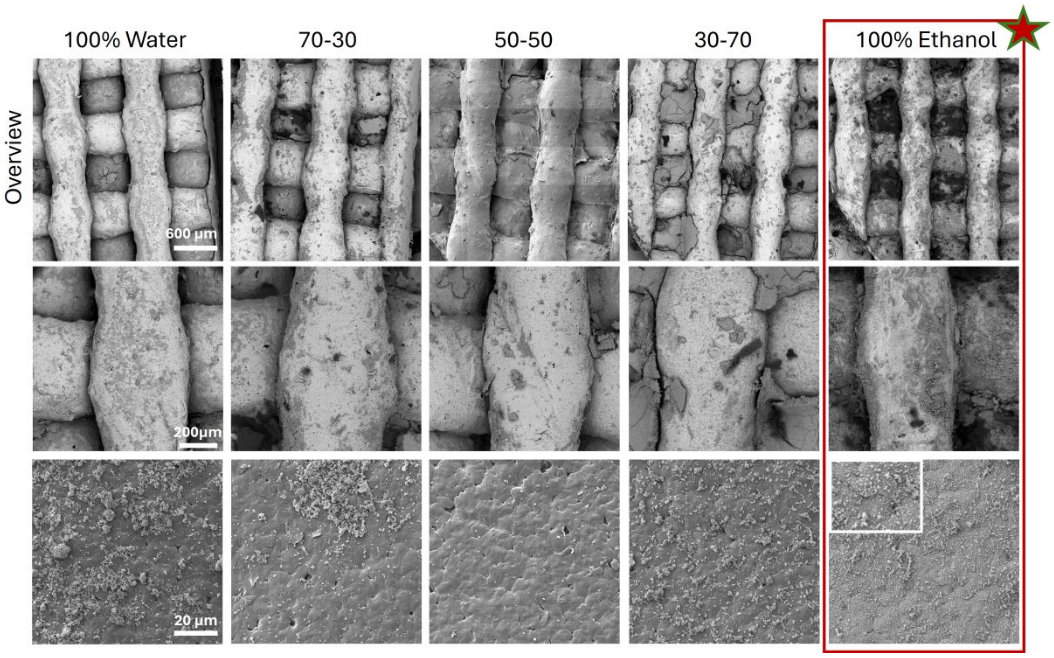

Figure S3. SEM images show the influence of type and composition of liquids on the efficiency of resuspending hydroxyapatite (HA) coatings on 3D printed scaffolds.

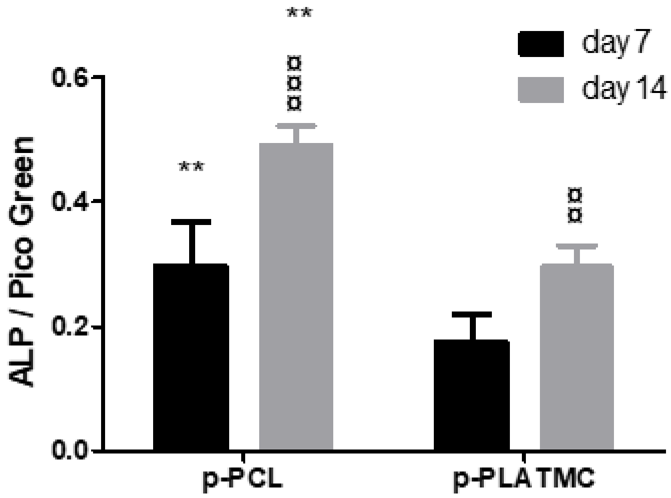

Figure S4. ALP activity comparison between p-PCL and p-PLATMC (material comparison).

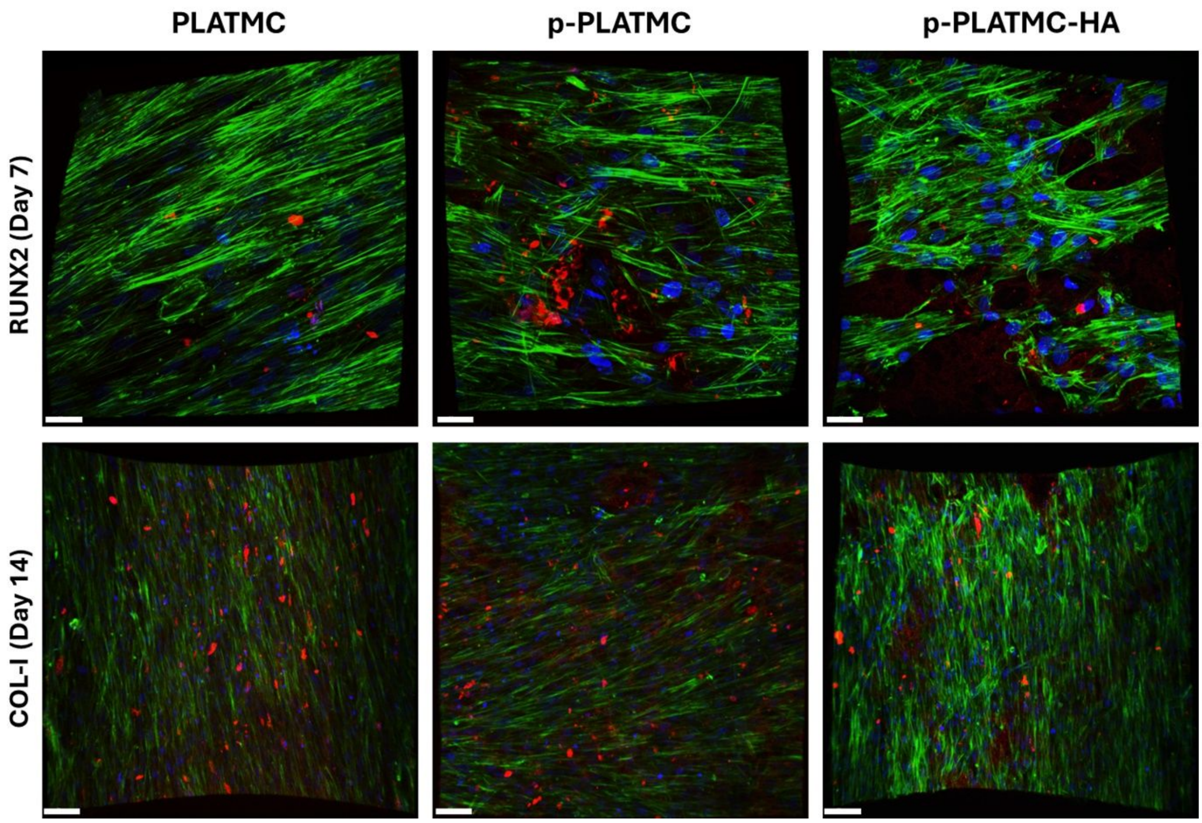

Figure S5. Immunofluorescence (IF) images showing RUNX2 (day 7) and COL-I (day 14) expression in hBMSCs. Red: RUNX2 or COL-I; green: phalloidin; blue: DAPI. Scale bars: 20 μm.

Immunofluorescence Staining

Seeded scaffolds were fixed in 4% paraformaldehyde (PFA) for 10 minutes at RT and then permeabilized with 0.1% Triton X-100 in PBS for 10 minutes at RT. To block nonspecific binding, 10% normal goat serum (NGS) (ab7481, Abcam, UK) with 1% Bovine Serum Albumin (BSA) (Sigma Aldrich/Merck) in PBS was applied for 2 hours at RT. Subsequently, the samples were incubated overnight at 4 ºC with RUNX2 rabbit polyclonal antibody (Product # PA5-82787, Invitrogen) at 1:200 dilution and Collagen type I recombinant rabbit monoclonal antibody (ST58-04) (Product # MA5-32178) at 1:200 dilution in diluted blocking solution (1% NGS with 1% BSA in PBS). Following primary antibody incubation, Goat anti-Rabbit IgG secondary antibody (Alexa Fluor™ 647, Invitrogen, ThermoFisher Scientific, MA, USA) was applied at 1:500 dilution in the blocking solution for 1 hour at RT to label the primary antibodies. Filamentous actin (F-actin) and nuclei were counterstained with Phalloidin Alexa Fluor 488 (1:250; A12379, ThermoFisher Scientific) and 4′,6-diamidino-2-phenylindole (DAPI, 1:5000; 62247, ThermoFisher Scientific) for 1 hour at RT. After staining, the samples were washed five times for 5 minutes each with PBS. Imaging was performed using an Andor Dragonfly 5050 high-speed confocal microscope and Fusion software (Oxford Instruments, Abingdon, UK). Images were acquired with a resolution of 1024 × 1024 using a high-speed iXon 888 Life EMCCD camera.

Calculations of NaCl-Based Porosity in 3D Printed Scaffolds

$$Volume\ of\ scaffold\ (V_{\sigma}^{s}) = Volume\ of\ PCL\ (V_{PCL}^{s}) + Volume\ of\ NaCl\ leached\ (V_{NaCl}^{s})$$

$$V_{\sigma}^{s} = V_{PCL}^{s} + V_{NaCl}^{s} \ldots (S1)$$

$$Microporosity\ (\emptyset_{\mu}) = \frac{Volume\ of\ NaCl\ Leached}{Volume\ of\ scaffold} = \frac{V_{NaCl}^{s}}{V_{\sigma}^{s}} = \frac{V_{NaCl}^{s}}{V_{PCL}^{s} + V_{NaCl}^{s}}$$

$$\emptyset_{\mu} = \frac{V_{NaCl}^{s}}{V_{PCL}^{s} + V_{NaCl}^{s}} \ldots (S2)$$

The volume of scaffold, polymer and salt seem unknown but can be derived or calculated via weight.

$$Weight\ of\ scaffold\ (w_{\sigma}^{s}) = Weight\ of\ PCL\ (w_{PCL}^{s}) + Weight\ of\ NaCl\ leached\ (w_{NaCl}^{s})$$

$$w_{\sigma}^{s} = w_{PCL}^{s} + w_{NaCl}^{s} \ldots (S3)$$

w_{NaCl}^{s} *The amount of NaCl leached from the scaffold can be found subtracting scaffold dry weight after leaching (=dry polymer weight) from the weight of before leaching (=weight of polymer + salt). We also know that the weight of scaffold before leaching is summation of weight of polymer and salt.*

$$w_{polymer+NaCl}^{s} = w_{dry,\ unleached}^{s} \ldots (S4)$$

$$w_{polymer}^{s} = w_{dry,leached}^{s} \ldots (S5)$$

$$w_{NaCl}^{s} = w_{dry,\ unleached}^{s} - w_{dry,leached}^{s} \ldots (S6)$$

$$w_{NaCl}^{s} = w_{polymer+NaCl}^{s} - w_{polymer}^{s} \ldots (S7)$$

Porosity is the direct function of volume not weight. Therefore, the relation should be based on the ratio of volumes. Volume can be rewritten via knowns of volume-weight-density relation: $V = \frac{w}{\rho}$

$$V_{NaCl}^{s} = \frac{w_{NaCl}^{s}}{\rho_{NaCl}}\ \&\ V_{polymer}^{s} = \frac{w_{polymer}^{s}}{\rho_{polymer}} \ldots (S8)$$

$$\emptyset_{\mu} = \frac{V_{NaCl}^{s}}{V_{PCL}^{s} + V_{NaCl}^{s}} \ldots (S9)$$

$$\emptyset_{\mu} = \frac{\frac{w_{NaCl}^{s}}{\rho_{NaCl}}}{\frac{w_{NaCl}^{s}}{\rho_{NaCl}} + \frac{w_{polymer}^{s}}{\rho_{polymer}}}; \ldots (S10)$$

$$if\ \frac{w_{NaCl}^{s}}{w_{polymer}^{s}} = 1;\ then\ \emptyset_{\mu}(theoretical) = \frac{\rho_{polymer}}{\rho_{NaCl} + \rho_{polymer}} \ldots (S11)$$
